# Artesunate Inhibits Neointimal Hyperplasia by Promoting IRF4 Associated Macrophage Polarization

**DOI:** 10.1002/advs.202408992

**Published:** 2025-03-24

**Authors:** Jinlin Miao, Yule Yong, Zhaohui Zheng, Kui Zhang, Wei Li, Jiayi Liu, Siyi Zhou, Juan‐juan Qin, Haoyang Sun, Yatao Wang, Xianghui Fu, Xing Luo, Siyu Chen, Zhi‐Gang She, Jingjing Cai, Ping Zhu

**Affiliations:** ^1^ Department of Clinical Immunology of Xijing Hospital and Department of Cell Biology of National Translational Science Center for Molecular Medicine Fourth Military Medical University Xi'an 710032 China; ^2^ Department of Cardiology Renmin Hospital Wuhan University Wuhan 430060 China; ^3^ Institute of Model Animal Wuhan University Wuhan 430071 China; ^4^ School of Basic Medical Science Wuhan University Wuhan 430071 China; ^5^ Department of Geriatrics Zhongnan Hospital Wuhan University Wuhan 430070 China; ^6^ Department of Cardiology The Third Xiangya Hospital Central South University Changsha 410013 China

**Keywords:** artesunate, IRF4, macrophages, neointimal hyperplasia, restenosis

## Abstract

Vascular restenosis is a serious clinical issue initiated and aggravated by macrophage inflammation, with no effective treatments available, in cardiovascular and autoimmune diseases. However, the untapped mechanisms and new targets that can regulate macrophage polarization and vascular restenosis remain elusive. The research identifies interferon regulatory factor 4 (IRF4) expression as crucial in macrophage polarization during arterial restenosis. Myeloid‐specific *Irf4* deficiency and overexpression experiments showed that IRF4 promoted M2 macrophage polarization, inhibited M1 macrophage transitions, and disrupted the interaction between macrophages and vascular smooth muscle cells to reduce neointimal hyperplasia by directly upregulating krüppel like factor 4 (KLF4) expression. Artesunate, an FDA‐approved drug, is screened as a potent activator of IRF4 expression in M2 polarization, and its treatment attenuated arterial restenosis in rodents and non‐human primates. The findings reveal a significant protective role of IRF4 in the development of neointimal hyperplasia by regulating macrophage polarization, and artesunate may be proposed as a novel therapy for vascular restenosis.

## Graphical abstract

This study shows that macrophage interferon regulatory factor 4 (IRF4) improved arterial injury‐induced neointimal hyperplasia by promoting of M2 polarization via up‐regulating krüppel‐like factor 4 (KLF4) in rodent and nonhuman primate models. Notably, artesunate is identified as a potent inducer of IRF4 in macrophages, and proposed as a promising novel therapy for vascular restenosis.

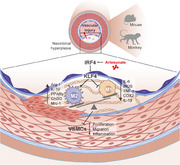

## 1 Introduction

Cardiovascular disease (CVD) is the leading cause of death and disability worldwide.^[^ ^1^ ^]^ Atherosclerosis, the primary pathological basis of CVD, is a chronic inflammatory disease that can be initiated and accelerated by local and systemic inflammation.^[^ ^2^ ^]^ For instance, autoimmune rheumatic diseases had an increased risk and mortality for cardiovascular events.^[^ ^3^ ^]^ Vascular interventions, including intravascular stenting, endarterectomy, and angioplasty, are the mainstays for treating atherosclerotic occlusion.^[^ ^4^ ^]^ However, postoperative arterial restenosis and the need for repeated revascularization still occur in many patients.^[^ ^5^ ^]^ While numerous drugs and drug‐eluting stents have been developed to mitigate this problem, their long‐term efficacy remains unsatisfactory, and the precise mechanisms and regulators contributing to restenosis are unclear. This ongoing challenge highlights the need for further investigation into the underlying mechanisms of restenosis to improve clinical outcomes.

Despite the extensive focus on vascular smooth muscle cells (VSMCs), mounting evidences indicate that inflammation response is a critical driver of restenosis occurrence and progression.^[^ ^6^ ^]^ Recent clinical trials have suggested that canakinumab^[^ ^7^ ^]^ and colchicine,^[^ ^8^ ^]^ with its unique anti‐inflammatory efficacy, are effective in reducing the risks of cardiovascular events and restenosis in patients with post‐intravascular stenting.^[^ ^9^, ^10^ ^]^ Macrophages, as primary inflammatory cells, significantly impact the inflammation in CVD and regulate aggressive neointimal proliferation, thereby contributing to restenosis by expressing specific phenotypes.^[^ ^11^, ^12^ ^]^ During the formation of vascular restenosis, macrophages typically polarize to the classically activated M1 phenotype, secreting proinflammatory cytokines that promote VSMC proliferation and migration.^[^ ^13^ ^]^ Conversely, alternatively activated M2 macrophages exhibit anti‐inflammatory properties, leading to a significant reduction in vascular restenosis.^[^ ^14^ ^]^ Consequently, it is crucial to elucidate the regulatory mechanisms of macrophage polarization to vascular injury, and further regulating macrophage polarization maybe a promising strategy to alleviate vascular inflammation and restenosis.

Within the hierarchical molecular network controlling macrophage polarization, several transcription factors have been identified to significantly impact the macrophage phenotypes.^[^ ^15^, ^16^ ^]^ Interferon regulatory factors (IRFs) are hub transcription factors that modulate immune responses in fighting viral infection.^[^ ^17^, ^18^ ^]^ In addition to the N‐terminal DNA‐binding domain that binds a consensus recognition sequence, most IRFs contain a protein interaction domain that allows for interaction with other IRF family members or transcription factors for diverse cellular functions.^[^ ^17^, ^18^ ^]^ For instance, IRF4 is constitutively expressed in macrophages and histone demethylation of the IRF4 promoter contributes to the M2 macrophage polarization under helminth parasite conditions.^[^ ^19^, ^20^ ^]^ However, given that the phenotype and function of macrophages differ in specific diseases, the precise role of IRF4 in governing macrophage phenotypes and the subsequent inflammatory response during vascular restenosis has yet to be clarified. In the present study, we explored the regulatory effect of IRF4 on macrophage polarization during vascular restenosis. Importantly, through high‐throughput screening analysis combined with in vitro and in vivo experiments, we investigated the protective effects of selected drugs against restenosis in both mouse and nonhuman primate models.

## 2 Results

### 2.1 Identification of IRF4 as a Regulator of Neointimal Hyperplasia and Macrophage Polarization

**Figure 1:**
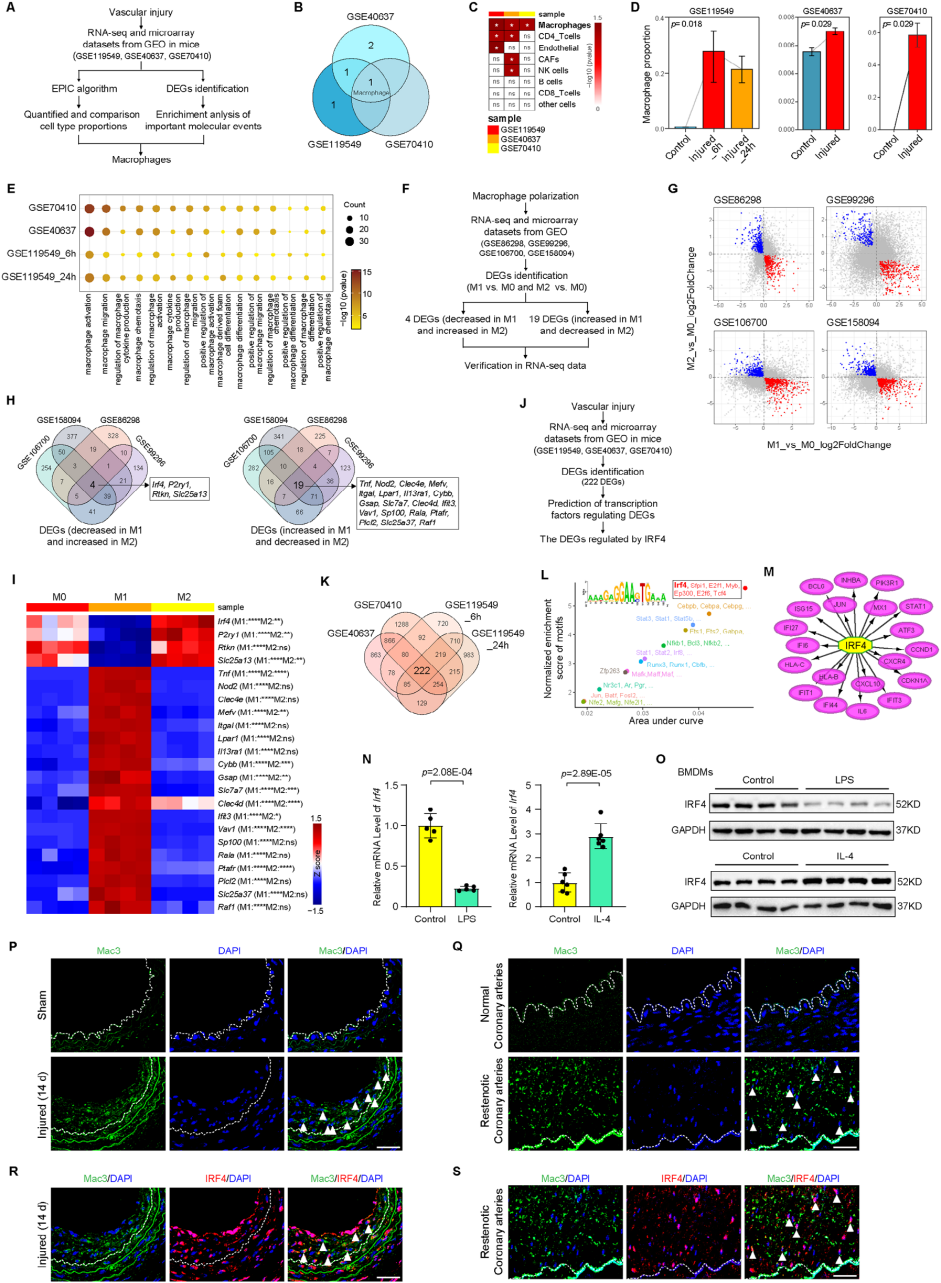
IRF4 is a regulator of neointimal hyperplasia and macrophage polarization. A) Flowchart for bioinformatics analysis of publicly available data from GEO databases of vascular injury in mice. B) Venn diagram showing the number of cells with significantly different abundances from the GSE119549, GSE40637, and GSE70410 datasets by the EPIC algorithm. C) Difference in predicted cell proportions between the vascular injury and control groups in GEO datasets. *p < 0.05, vascular injury versus control group. D) Difference in macrophage proportion between the vascular injury and control groups in GEO datasets. E) GO‐biological process enrichment analysis of the 222 common DEGs. F) Flowchart for analysis of macrophage polarization from GEO databases. G) Volcano plot showing the DEGs in 4 GEO datasets of macrophage polarization. Blue dots represent the genes with significantly downregulated expression in M1 and upregulated expression in M2 macrophages. Red dots represent the genes with significantly upregulated expression in M1 and downregulated expression in M2 macrophages. H) Venn diagram showing the common DEGs shared by the 4 GEO datasets. I) Heatmaps showing the expression of 23 DEGs identified in (H) in the BMDMs of mice with M1 (LPS) and M2 (IL‐4) compared to M0 (Vehicle) macrophages from our RNA‐seq dataset. *p < 0.05, **p < 0.01, ***p < 0.001, ****p < 0.0001 versus M0 group. J) Flowchart for transcription factor (TF) prediction of the 222 common DEGs from vascular injury databases. K) Venn diagram showing the common DEGs in GEO databases of vascular injury (GSE119549, GSE40637, and GSE70410). L) The TFs of the hub DEGs predicted by iRegulon analysis. M) Vascular injury‐related DEGs regulated by IRF4. N) Relative IRF4 mRNA expression levels in mouse BMDMs upon LPS and IL‐4 treatment. *n* = 5 to 6 per group. O) Western blot analysis of IRF4 protein levels in mouse BMDMs upon LPS and IL‐4 treatment. P,Q) Immunofluorescence staining of Mac3 in sections of mouse wire‐induced carotid neointima (P) and human coronary arteries (Q). Arrowheads indicate Mac3‐positive macrophages. Scale bar = 25 µm. R,S) Immunofluorescence costaining of IRF4 (red) with Mac3 (green) in sections of mouse wire‐induced carotid neointima (R) and human coronary arteries (S). Arrowheads indicate Mac3‐ and IRF4‐positive macrophages. Scale bar = 25 µm. ns, not significant. EPIC, Estimating the Proportion of Immune and Cancer cells. DEGs, differentially expressed genes. LPS, lipopolysaccharides. Data information: The data with error bars are presented as mean ± SD. One‐way ANOVA followed by Bonferroni's test (D, I) and Two‐tailed Student's t‐test (D, N) are used.

To identify the key regulators potentially associated with post‐injury remodeling in the vasculature, a composite analysis was performed to explore the critical molecular changes by mining the public Gene Expression Omnibus (GEO) datasets obtained from mouse aortic tissues in response to vascular injuries (**Figure** **1**A). By Estimating the Proportion of Immune and Cancer cells (EPIC) algorithm, we performed the analysis of three independent public GEO datasets and found that macrophages were the only common cell type in injured tissues which are significantly higher in proportions than in control tissues (Figure 1B–D). Interestingly, enrichment analysis also identified macrophage‐related biological processes as the main events across these GEO datasets (Figure 1E). Among them, macrophage polarization is closely linked to macrophage activation, migration, and cytokine production, and is involved in vascular restenosis.^[^ ^13^, ^21^ ^]^ To further delineate the key molecules regulating macrophage polarization, we analyzed 4 separate public datasets of polarized macrophages and identified a total of 23 shared differentially expressed genes (DEGs) in M1 and M2 macrophages, as illustrated in the Venn diagram (Figure 1F–H). These 23 DEGs were then verified with our RNA‐seq data of primary mouse bone marrow‐derived macrophages (BMDMs), and as compared to M0 (Vehicle) cells, IRF4 was the most significantly downregulated gene in lipopolysaccharides (LPS)‐induced M1 cells, but its expression was upregulated in interleukin‐4 (IL‐4)‐induced M2 macrophages (Figure 1I). Furthermore, we performed iRegulon analysis of the common DEGs to predict the key transcription factors (TFs) involved in vascular injury (Figure 1J). As illustrated in the Venn diagram, 222 DEGs were shared among vascular injury‐related GEO datasets (Figure 1K). Additionally, we identified IRF4 as the top‐ranked TF (Figure 1L,M), indicating that IRF4 is a potential key regulator involved in macrophage polarization in response to vascular injury.

Consistent with above‐mentioned results of bioinformatics analysis, the expression of IRF4 was significantly decreased in primary LPS‐stimulated M1 macrophage, but increased in IL‐4‐stimulated M2 phenotype (Figure 1N,O; Figure S1A, Supporting Information). Furthermore, abundant IRF4‐positive macrophage infiltration was observed in the neointima of mouse carotid arteries (Figure 1P,R) and human coronary arteries (Figure 1Q,S) by immunofluorescence microscopy. In addition, the levels of infiltration M1 macrophages (marked with CD80) was higher than that of M2 macrophages (marked with CD206) in neointima of mouse arteries post injured, and IRF4 expression was significantly increased in M2 macrophages (Figure S1B, Supporting Information). All these data implicate IRF4 as a potential key regulatory factor in macrophage polarization and neointima formation post‐injury.

### 2.2 IRF4 Regulates Macrophage Polarization and its Crosstalk with VSMCs

**Figure 2:**
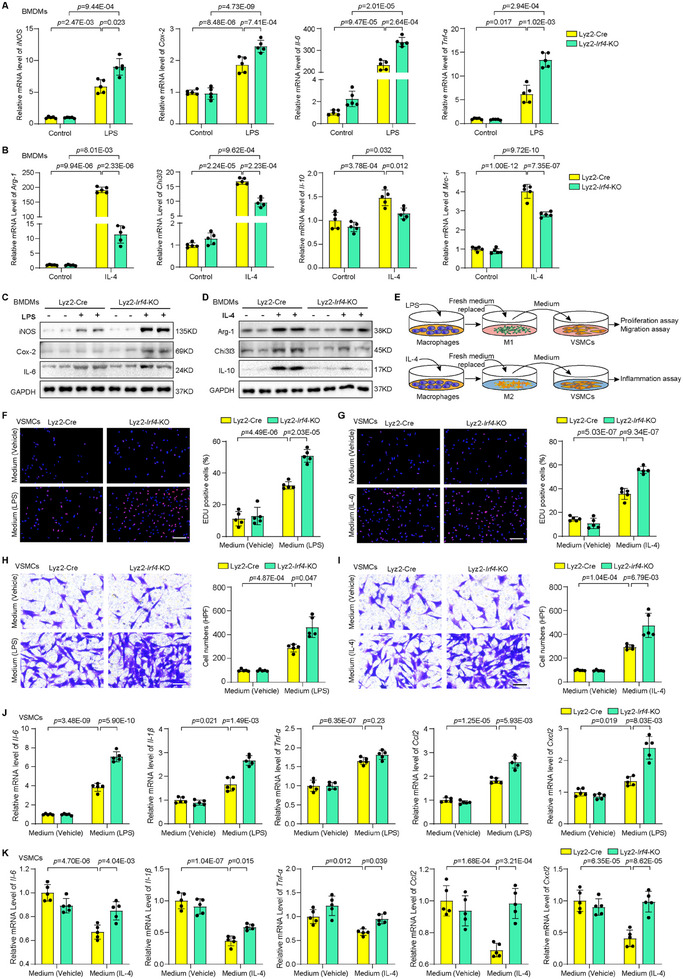
IRF4 regulates macrophage polarization and smooth muscle cell activity. A) Relative mRNA expression levels of M1 macrophage markers in BMDMs from Lyz2‐Cre and Lyz2‐*Irf4*‐KO mice upon LPS treatment. *n* = 5 per group. B) Relative mRNA expression levels of M2 macrophage markers in BMDMs from Lyz2‐Cre and Lyz2‐*Irf4*‐KO mice upon IL‐4 treatment. *n* = 5 per group. C,D) Western blot analysis of M1 macrophage (C) or M2 macrophage (D) marker expression from mouse BMDMs upon LPS and IL‐4 treatment. E) Schematic diagram of the collection of conditioned macrophage medium and smooth muscle cell activity analysis. F,G) EdU incorporation was performed to assess VSMC proliferation in conditioned medium collected from cultured BMDMs of Lyz2‐Cre and Lyz2‐*Irf4*‐KO upon DMSO (Vehicle), LPS (F) or IL‐4 (G) treatment. Scale bar = 200 µm. *n* = 5 per group. H,I) The number of migrated VSMCs detected using the Transwell migration assay induced by coculturing with conditioned medium of Lyz2‐Cre and Lyz2‐*Irf4*‐KO BMDMs treated with DMSO (Vehicle), LPS (H) or IL‐4 (I) in the bottom. Scale bar = 100 µm. *n* = 5 per group. J,K) Relative mRNA expression levels of smooth muscle cell activation markers in VSMCs cocultured with conditioned medium from Lyz2‐Cre and Lyz2‐*Irf4*‐KO BMDMs upon DMSO (Vehicle), LPS (J) or IL‐4 (K) treatment. *n* = 5 per group. Data information: The data with error bars are presented as mean ± SD. One‐way ANOVA followed by Bonferroni's test (A, B, F, K, G, J) and Tamhane's T2 test (A, B, H, I, J) are used.

To evaluate IRF4 function in macrophage polarization in vivo, we generated myeloid‐specific *Irf4* knockout (Lyz2‐*Irf4*‐KO) mice using Lyz2‐Cre‐mediated deletion of the *Irf4* gene (Figure S2A, Supporting Information). Western blotting showed that IRF4 expression was more significantly suppressed in BMDMs prepared from Lyz2‐*Irf4*‐KO mice compared to that in control Lyz2‐Cre mice (Figure S2B, Supporting Information). The LPS‐stimulated M1 macrophage‐associated genes, including *iNOS, Cox‐2, Il‐6*, and *Tnf‐α*, were further induced at the mRNA level in the Lyz2‐*Irf4*‐KO BMDMs compared to that in the control BMDMs (**Figure** **2**A). In contrast, the IL‐4‐induced M2 macrophage‐associated genes *Arg‐1, Chi3l3, Il‐10*, and *Mrc‐1* were significantly attenuated at the mRNA level in the Lyz2‐*Irf4*‐KO BMDMs (Figure 2B). Consistently, Western blotting showed a similar impact of *Irf4* KO on M1/M2 signature genes at the protein level in BMDMs (Figure 2C,D; Figure S2C,D, Supporting Information).

Next, we evaluated whether IRF4‐mediated macrophage polarization could affect VSMC proliferation, migration, and inflammation. Supernatants from Lyz2‐Cre and Lyz2‐*Irf4*‐KO BMDMs stimulated by LPS or IL‐4 were collected and used as conditioned media to treat VSMCs, followed by EdU assay and transwell chamber assay to measure proliferation and migration (Figure 2E). The LPS‐conditioned media from Lyz2‐*Irf4*‐KO BMDMs further exacerbated VSMC proliferation and migration compared to the media from control BMDMs (Figure 2F,H). And the conditioned medium from IL‐4‐induced M2 macrophages promoted VSMC proliferation and migration, which is further enhanced in Lyz2‐*Irf4*‐KO BMDMs (Figure 2G,I). Additionally, LPS‐conditioned medium increased the mRNA levels of proinflammatory cytokines and chemokines (*Il‐6, Il‐1β, Tnf‐α, Ccl2*, and *Cxcl2*) in VSMCs, with this increase further amplified in Lyz2‐*Irf4*‐KO BMDMs (Figure 2J). The expression of proinflammatory cytokines and chemokines were significantly decreased in VSMCs treated with IL‐4‐conditioned medium from BMDMs, but markedly increased by IL‐4‐conditioned medium from Lyz2‐*Irf4*‐KO BMDMs (Figure 2K). Further experiments showed that IRF4‐mediated polarization of M1 and M2 macrophages primarily regulate VSMC proliferation and migration via IL‐6 and IL‐10 secretion, respectively (Figure S2E‐I, Supporting Information). These results revealed the impact of IRF4 expression on macrophage polarization and the role of crosstalk signaling from macrophages to VSMCs in determining their proliferation, migration, and inflammatory profiles.

### 2.3 IRF4 Regulates Macrophage Polarization In Vivo

**Figure 3:**
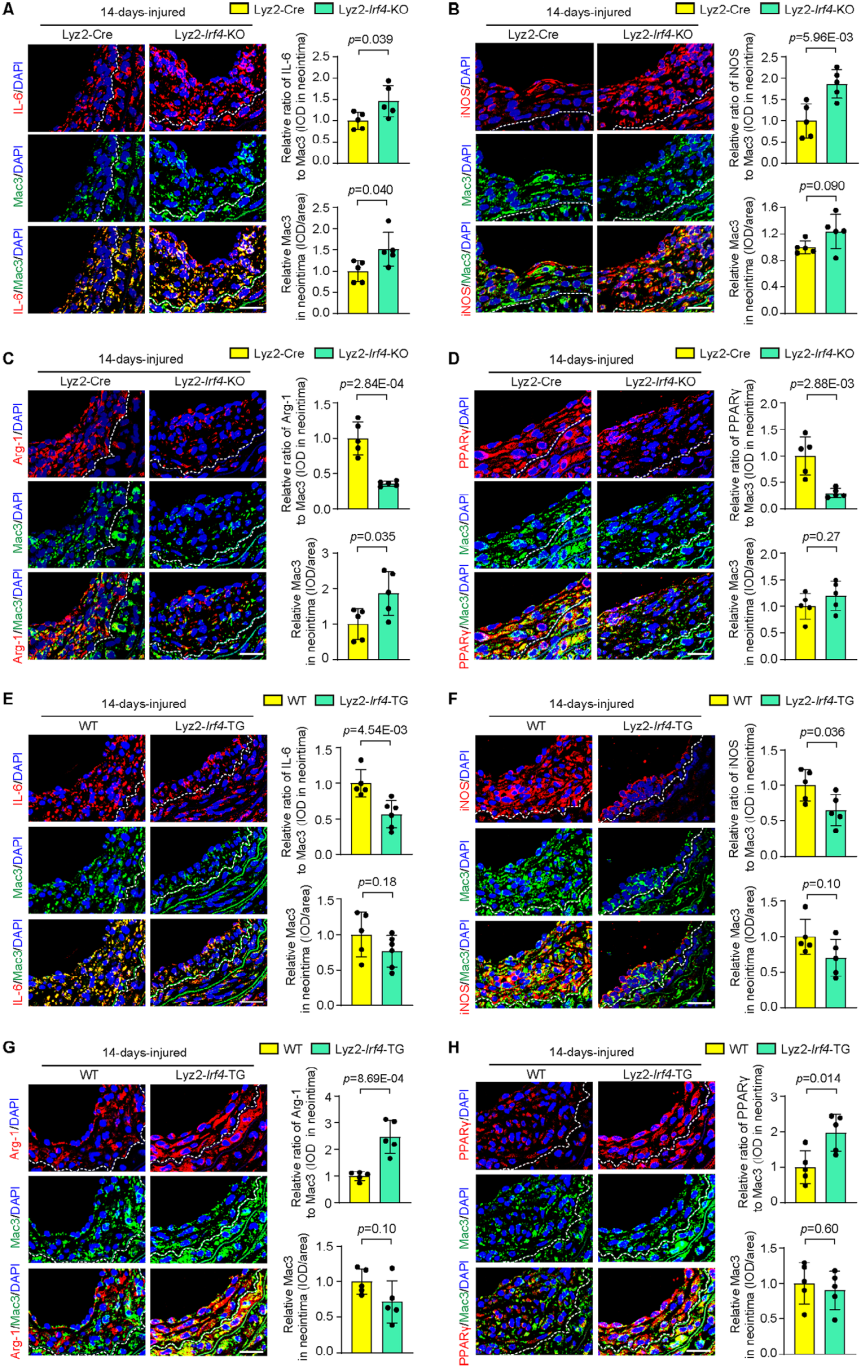
IRF4 regulates macrophage polarization in vivo. A–D) Immunofluorescence costaining of IL‐6 (A), iNOS (B), Arg‐1 (C), or PPARγ (D) (red) with Mac3 (green) in sections of wire‐induced carotid neointima from Lyz2‐Cre and Lyz2‐*Irf4*‐KO mice. *n* = 5 per group. E–H) Immunofluorescence costaining of IL‐6 (E), iNOS (F), Arg‐1 (G), or PPARγ (H) (red) with Mac3 (green) in sections of wire‐induced carotid neointima from WT and Lyz2‐*Irf4*‐TG mice. *n* = 5 to 6 per group. Immunofluorescence data are graphically represented as the relative positive area of IL‐6, iNOS, Arg‐1, or PPARγ immunostaining normalized to Mac3 and the relative expression level of Mac3 in the neointima. All scale bar = 25 µm. IOD, Integrated Optical Density. Data information: The data with error bars are presented as mean ± SD. Two‐tailed Student's t‐test is used.

The function of macrophage IRF4 in vascular injury was evaluated in the mouse carotid artery following wire injury. Based on immunofluorescence staining, IL‐6 and iNOS expression in neointimal macrophages was significantly elevated in the Lyz2‐*Irf4*‐KO mice at day 14 after wire injury (**Figure** **3**A,B). In contrast, the expression of the immunosuppressive genes Arg‐1 and PPARγ was significantly suppressed in neointima macrophages in Lyz2‐*Irf4*‐KO mice compared to that in control mice (Figure 3C,D). To further demonstrate the regulatory role of IRF4 in macrophage polarization, we generated myeloid‐specific *Irf4*‐overexpressing transgenic mice (Lyz2‐*Irf4*‐TG) by using a transgenic construct containing mouse *Irf4* cDNA under the control of a myeloid‐specific promoter of the mouse Lyz2 gene (Figure S2J, Supporting Information). IRF4 expression in BMDMs prepared from Lyz2‐*Irf4*‐TG mice was significantly upregulated in comparison to that in BMDMs prepared from wild‐type (WT) controls (Figure S2K, Supporting Information). A significant decrease in the expression of IL‐6 and iNOS (Figure 3E,F) and a significant increase in Arg‐1 and PPARγ (Figure 3G,H) were observed in neointimal macrophages in Lyz2‐*Irf4*‐TG mice at day 14 post‐injury. Therefore, the in vivo loss‐ and gain‐of‐function analyses both support the notion that IRF4 expression promotes macrophage polarization to the M2 phenotype in the mouse carotid artery following wire injury.

### 2.4 IRF4 Inhibits Arterial Neointima Formation in Mice and Nonhuman Primates

**Figure 4:**
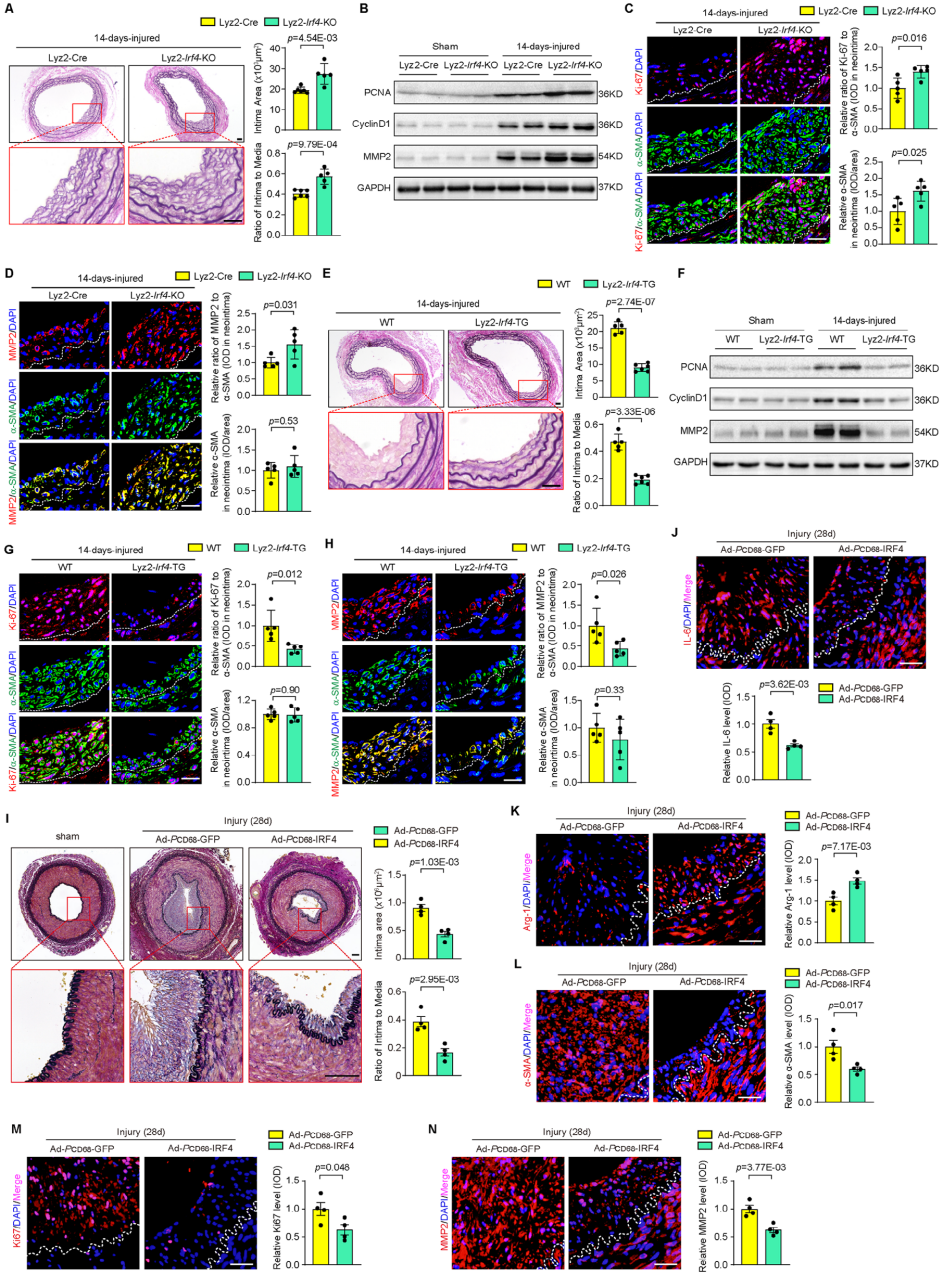
IRF4 inhibits arterial neointima formation in mice and nonhuman primates. A) Elastica van Gieson (EVG)‐stained sections showing the intimal areas in Lyz2‐Cre and Lyz2‐*Irf4*‐KO mice after wire‐induced carotid injury. Quantified intimal area and the intima‐to‐media (I/M) ratio. *n* = 5 to 6 per group. Scale bar = 25 µm. B) Western blot analysis of PCNA, CyclinD1 and MMP2 proteins in the arteries of Lyz2‐Cre and Lyz2‐*Irf4*‐KO mice after sham or at 14 days post‐injury. C,D) Immunofluorescence costaining of Ki67 (C) or MMP2 (D) (red) with α‐SMA (green) in arteries from Lyz2‐Cre and Lyz2‐*Irf4*‐KO mice. *n* = 5 per group. Scale bar = 25 µm. E) EVG‐stained sections demonstrating the intimal areas in WT and Lyz2‐*Irf4*‐TG mice after wire‐induced carotid injury. *n* = 5 to 6 per group. Scale bar = 25 µm. F) Western blot analysis of PCNA, CyclinD1 and MMP2 proteins in the arteries of WT and Lyz2‐*Irf4*‐TG mice after sham or at 14 days post‐injury. G,H) Immunofluorescence costaining of Ki67 (G) or MMP2 (H) (red) with α‐SMA (green) in arteries from WT and Lyz2‐*Irf4*‐TG mice. *n* = 5 per group. Scale bar = 25 µm. I) EVG‐stained sections demonstrating the intimal areas in sham monkeys and balloon‐induced femoral artery injury from Ad‐*P*CD68‐GFP and Ad‐*P*CD68‐IRF4 monkeys. Quantified intimal area and the I/M ratio. *n* = 4 per group. Scale bar = 100 µm. J–N) Immunofluorescence staining of IL‐6 (J), Arg‐1 (K), α‐SMA (L), Ki67 (M), and MMP2 (N) in arteries from Ad‐*P*CD68‐GFP and Ad‐*P*CD68‐IRF4 monkeys. *n* = 4 per group. Scale bar = 20 µm. The mouse immunofluorescence data is graphically represented as the relative positive area of Ki67 or MMP2 immunostaining normalized to α‐SMA and the relative expression level of α‐SMA in the neointima. The monkey immunofluorescence data is graphically represented as the relative expression level of these markers in the neointima. IOD, Integrated Optical Density. Data information: The data with error bars are presented as mean ± SD. Two‐tailed Student's t‐test is used.

To identify the pathophysiological role of macrophage IRF4 in neointima formation, wire injury‐induced carotid arterial restenosis was evaluated in Lyz2‐*Irf4*‐KO, Lyz2‐*Irf4*‐TG, and their corresponding control mice. The severity of restenosis was determined by the neointimal area and the intima/media (I/M) ratio. The Lyz2‐*Irf4*‐KO mice showed exacerbated restenosis compared with the Lyz2‐Cre mice at day 14 after wire injury (**Figure** **4**A). As shown by Western blotting, the expression of proliferation markers (PCNA and CyclinD1) and matrix metalloproteinase in remodeling of the vasculature (MMP2) were further upregulated in injured vessels in Lyz2‐*Irf4*‐KO mice compared to the control mice (Figure 4B; Figure S2L, Supporting Information). In addition, molecular markers for cell proliferation (Ki67) and remodeling (MMP2) were further increased in neointima smooth muscle cells in the Lyz2‐*Irf4*‐KO mice compared with that in the controls (Figure 4C,D). In contrast, neointima formation in the carotid arteries was significantly attenuated in Lyz2‐*Irf4*‐TG mice compared with that in WT controls (Figure 4E). Western blotting showed that the expression of PCNA, CyclinD1 and MMP2 was also downregulated in the Lyz2‐*Irf4*‐TG mice compared to the WT mice after injury (Figure 4F; Figure S2M, Supporting Information). Consistently, Ki67 and MMP2 signals were suppressed in the neointima smooth muscle cells in the Lyz2‐*Irf4*‐TG mice compared to the WT mice following injury (Figure 4G,H). All these data suggest that myeloid‐specific *Irf4* exerts a protective effect on neointima formation in mice. Furthermore, to explore the clinical relevance of macrophage IRF4 in the treatment of vascular restenosis, we constructed a myeloid‐specific adenovirus vector carrying the full‐length IRF4 coding sequence driven by the CD68 promoter (Ad‐*P*CD68‐IRF4). The IRF4 adenovirus was injected intravascularly following the femoral artery injury in monkeys (Figure S3A, Supporting Information). Immunofluorescence staining revealed that IRF4 was specifically expressed in CD68‐positive macrophages in the transfected femoral artery (Figure S3B, Supporting Information). Importantly, the neointimal area and the I/M ratio in the femoral arteries were significantly reduced in Ad‐*P*CD68‐IRF4‐transfected monkeys compared to the control monkeys transfected with Ad‐*P*CD68‐GFP (Figure 4I). Immunofluorescence staining showed a lower IL‐6 (M1 phenotype marker) expression (Figure 4J) and a higher Arg‐1 (M2 phenotype marker) expression (Figure 4K) in the injured vessels from Ad‐*P*CD68‐IRF4‐treated monkeys than in those from Ad‐*P*CD68‐GFP‐treated monkeys at day 28 post‐injury. Furthermore, Ad‐*P*CD68‐IRF4 treatment reduced α‐smooth muscle actin (α‐SMA), Ki67 and MMP2 expression levels in the injured vessels (Figure 4L–N). These data indicate that IRF4 expression in macrophages regulates macrophage polarization and inhibits neointima formation in nonhuman primates.

### 2.5 KLF4 Mediates IRF4‐Regulated Macrophage Polarization and Neointimal Formation

**Figure 5:**
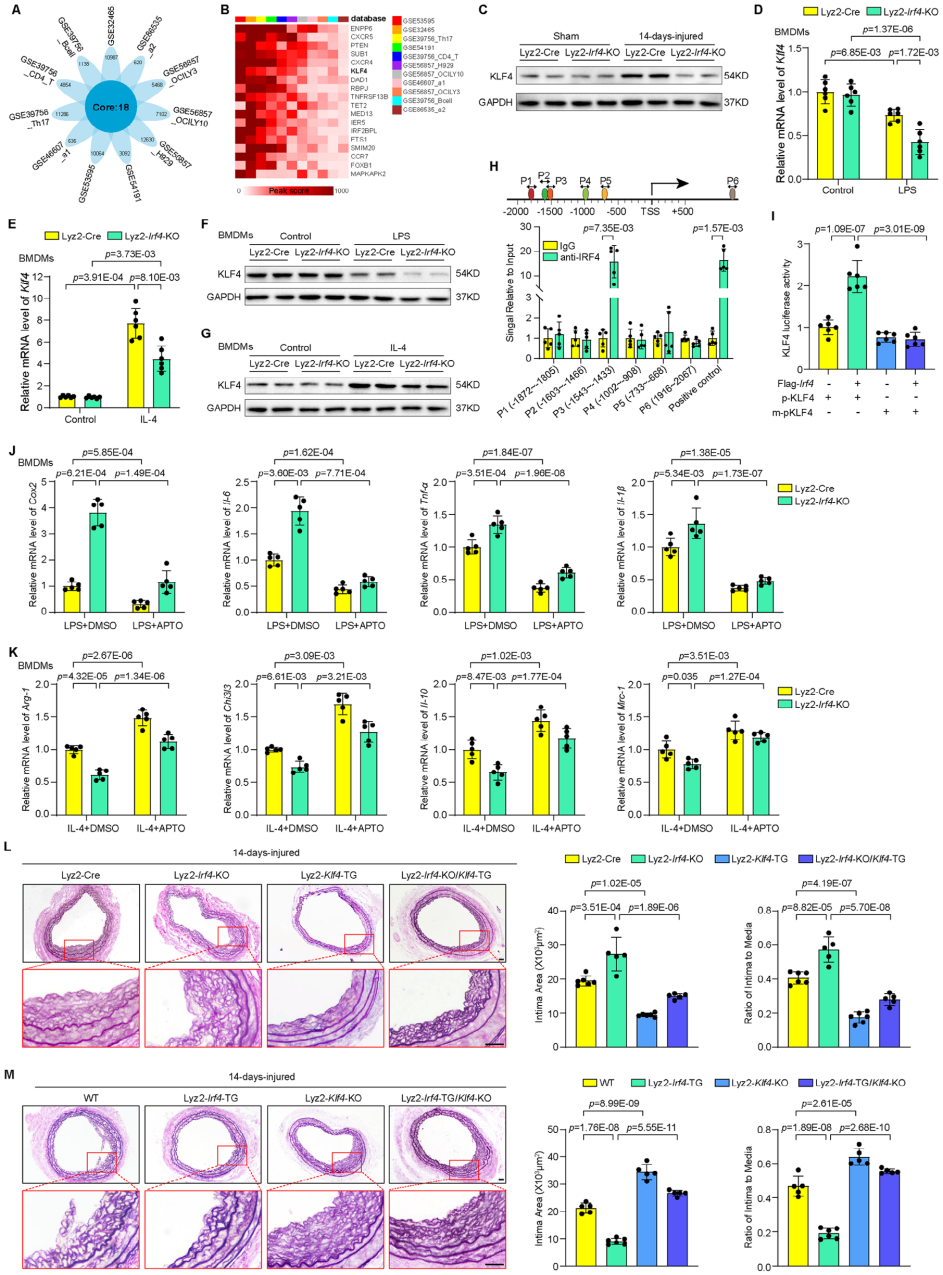
KLF4 mediates IRF4 regulation of macrophage polarization and neointimal formation. A) Venn diagram showing the core binding targets of IRF4 conserved across GEO ChIP‐Seq databases. B) Heatmaps showing the peak scores of 18 core binding targets identified in (A). C) Western blot analysis of KLF4 protein in the arteries of Lyz2‐Cre and Lyz2‐*Irf4*‐KO mice after sham or at 14 days post‐injury. D,E) Relative mRNA expression KLF4 level in BMDMs from Lyz2‐Cre and Lyz2‐*Irf4*‐KO mice upon LPS (D) or IL‐4 (E) treatment. *n* = 6 per group. F,G) Western blot analysis of KLF4 protein in BMDMs from Lyz2‐Cre and Lyz2‐*Irf4*‐KO mice upon LPS (F) or IL‐4 (G) treatment. H) Relative enrichment of IRF4 binding at the indicated putative interferon‐stimulating response elements as determined by ChIP assays. mIL12‐pro84 served as a positive control. *n* = 5 per group. I) Relative KLF4 promoter activity of BMDMs transfected with AdIRF4, pKLF4‐luciferase, and m‐pKLF4‐luciferase (IRF4 binding site pro110 deletion). *n* = 6 per group. J,K) Relative mRNA expression levels of M1 macrophage (J) or M2 macrophage (K) markers in BMDMs from Lyz2‐Cre and Lyz2‐*Irf4*‐KO mice upon DMSO or APTO‐253 (APTO) stimulation under LPS or IL‐4 treatment. *n* = 5 per group. L,M) EVG‐stained sections demonstrating the intimal areas in Lyz2‐Cre, Lyz2‐*Irf4*‐KO, Lyz2‐*Klf4*‐TG, and Lyz2‐*Irf4*‐KO/*Klf4*‐TG mice (L) and WT, Lyz2‐*Irf4*‐TG, Lyz2‐*Klf4*‐KO, and Lyz2‐*Irf4*‐TG/*Klf4*‐KO mice (M) after wire‐induced carotid injury. *n* = 5 to 6 per group. Scale bar = 25 µm. TSS, transcription start site. Data information: The data with error bars are presented as mean ± SD. One‐way ANOVA followed by Bonferroni's test (D, I, J, K, L, M) or Tamhane's T2 test (E, J, K), and Two‐tailed Student's t‐test (H) are used.

To further elucidate the mechanisms underlying the anti‐inflammatory effects of IRF4 in macrophage regulation and vascular restenosis, IRF4 chromatin immunoprecipitation (ChIP)‐seq data obtained from 11 public GEO datasets were integrated and dissected (**Figure** **5**A). A total of 18 core binding targets of IRF4 were identified in heatmap (Figure 5A,B). Among them, krüppel like factor 4 (KLF4) is a well‐established regulator of macrophage polarization and vascular neointima formation.^[^ ^22^, ^23^ ^]^ Western blot analysis showed a significant increase in KLF4 protein in the carotid arteries of Lyz2‐Cre mice 14 days after injury, but its level was significantly suppressed in Lyz2‐*Irf4*‐KO mice (Figure 5C; Figure S4A, Supporting Information). Further, the mRNA and protein levels of KLF4 were markedly decreased by LPS treatment in BMDMs from Lyz2‐Cre mice but were further reduced in BMDMs from Lyz2‐*Irf4*‐KO mice (Figure 5D,F; Figure S4B, Supporting Information). In contrast, KLF4 level was markedly elevated by IL‐4 treatment in BMDMs from Lyz2‐Cre mice, and this elevation was attenuated in BMDMs from Lyz2‐*Irf4*‐KO mice (Figure 5E,G; Figure S4C, Supporting Information). These data showed that inactivation of IRF4 suppressed KLF4 expression in macrophages. To determine the transcriptional regulation of *Klf4* by IRF4, we investigated IRF4 interaction with the *Klf4* promoter in BMDMs. ChIP assays demonstrated that a fragment −1543 to ≈−1433 bp (P3) from the transcription initiation site of the *Klf4* gene showed significant binding activity to IRF4 and contained a putative interferon‐stimulated response element (ISRE) (Figure 5H). A luciferase assay showed that *Klf4* promoter activity was induced by IRF4 overexpression in BMDMs (p‐KLF4), and mutation of the putative IRF4 binding sites in the *Klf4* promoter (m‐pKLF4) attenuated IRF4‐induced *Klf4* transcription (Figure 5I). Treatment of BMDMs with APTO‐253, an inducer of KLF4,^[^ ^24^ ^]^ significantly reversed the LPS‐induced *Klf4* suppression and further potentiated IL‐4‐induced *Klf4* expression (Figure S4D, E, Supporting Information). In addition, APTO‐253 treatment attenuated LPS‐induced upregulation of proinflammatory cytokine expression (Figure 5J) and potentiated IL‐4‐induced immunosuppressive gene expression (Figure 5K) in BMDMs from Lyz2‐*Irf4*‐KO mice, further supporting the role of KLF4 in macrophage phenotype transition.

Next, to investigate whether KLF4 in macrophages affects vascular restenosis in vivo, we generated a myeloid‐specific *Klf4* knockout mouse line (Lyz2‐*Klf4*‐KO) and a *Klf4* overexpressing transgenic mouse line (Lyz2‐*Klf4*‐TG) (Figure S5A‐D, Supporting Information). Then, myeloid‐specific Lyz2‐*Irf4*‐KO/*Klf4*‐TG mice and Lyz2‐*Irf4*‐TG/*Klf4*‐KO mice were created and tested (Figure S5E‐H, Supporting Information). Compared with the Lyz2‐Cre control group, neointima formation following wire injury was significantly inhibited in the Lyz2‐*Klf4*‐TG mice (Figure 5L). In addition, KLF4 expression also reversed the neointimal area and the I/M ratio in Lyz2‐*Irf4*‐KO/*Klf4*‐TG mice compared to Lyz2‐*Irf4*‐KO mice (Figure 5L). However, in the Lyz2‐*Irf4*‐TG/*Klf4*‐KO mice, the protective effects of macrophage IRF4 overexpression were eliminated by simultaneous KLF4 deletion (Figure 5M). Therefore, KLF4 is an essential downstream attributor for IRF4‐mediated protection against neointima formation.

### 2.6 Screening of Drugs Targeting IRF4 for Macrophage Polarization

**Figure 6:**
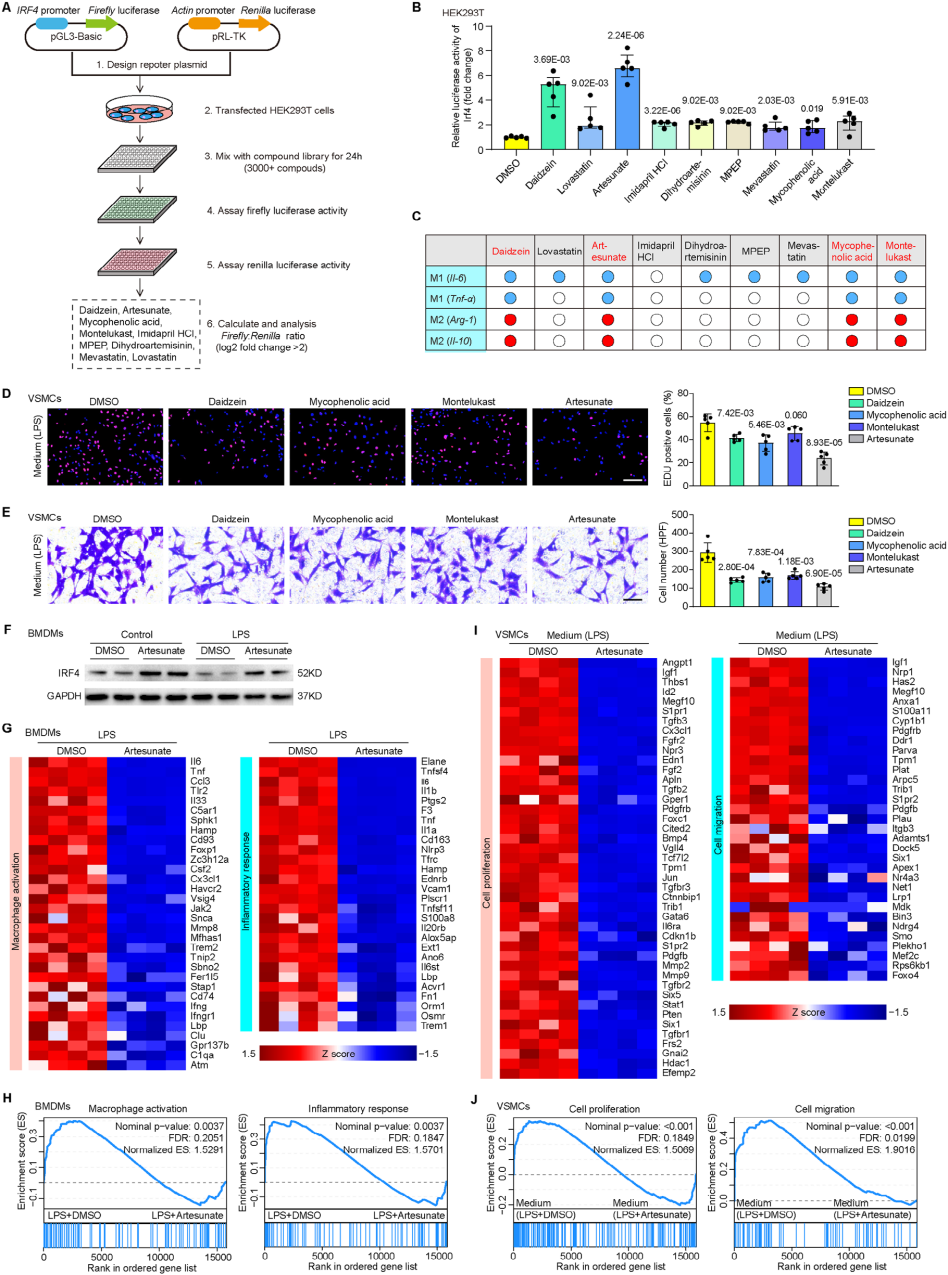
Screening drugs as potent regulators of macrophage polarization. A) Schematic diagram illustrating the experimental workflow of the dual‐luciferase‐based reporter assay kinome screen. B) The relative luciferase activity of Irf4 in HEK293T cells treated with 9 screened compounds. *n* = 5 per group. *p* value versus dimethyl sulfoxide (DMSO) group. C) The table shows the comprehensive effects of 9 compounds on regulating M1 (*Il‐6* and *Tnf‐α*) and M2 (*Arg‐1* and *Il‐10*) macrophage polarization. Blue dots represent a significant decrease compared with the control. Red dots represent a significant increase compared with the control. D) EdU incorporation was performed to assess VSMC proliferation in conditioned medium collected from cultured LPS‐treated BMDMs upon stimulation of daidzein, mycophenolic acid, montelukast and artesunate. *n* = 5 per group. *p* value versus DMSO group. Scale bar = 200 µm. E) The number of migrated VSMCs detected using the Transwell migration assay induced by coculturing with conditioned medium of LPS‐treated BMDMs upon compound stimulation in the bottom. *n* = 5 per group. *p* value versus DMSO group. Scale bar = 100 µm. F) Immunoblotting analysis of IRF4 protein levels in LPS‐treated BMDMs upon DMSO or artesunate stimulation. G,H) In LPS‐treated BMDMs stimulated with DMSO and artesunate, heatmaps show the expression profile of genes related to macrophage activation and the inflammatory response (G), and gene set enrichment analysis (GSEA) plots show the enrichment score of macrophage activation and inflammatory response gene sets based on RNA‐seq data (H). *n* = 4 per group. I,J) In VSMCs cocultured with conditioned medium of LPS‐treated BMDMs upon DMSO or artesunate stimulation, heatmaps showing the expression profile of genes related to cell proliferation and migration (I), GSEA plots showing the enrichment score of cell proliferation and migration gene sets based on RNA‐seq data (J). *n* = 4 per group. Data information: The data with error bars are presented as median ± interquartile range (B) or mean ± SD (D, E). Two‐tailed Student's t‐test (B, D, E) and Mann‐Whitney U test (B) are used.

Considering the protective effect of IRF4 on intimal hyperplasia, targeting upregulation of IRF4 is a potential therapeutic approach for preventing restenosis. Therefore, we screened a small molecule library containing 3008 FDA‐approved drugs using a highly sensitive dual‐luciferase‐based reporter assay, among which 9 compounds significantly improved IRF4 luciferase activity (**Figure** **6**A,B). The effects of screened compounds on IRF4 expression and macrophage polarization were further evaluated in BMDMs. Results showed that 9 compounds were able to upregulate *Irf4* levels in BMDMs (Figure S6A, Supporting Information). Concurrently, daidzein, artesunate, mycophenolic acid, and montelukast significantly inhibited M1 and promoted M2 polarization, as evidenced by their effects on reducing *Il‐6* and *Tnf‐α* (Figure 6C; Figure S6B, C, Supporting Information) while increasing *Arg‐1* and *Il‐10* mRNA levels (Figure 6C; Figure S6D, E, Supporting Information). Moreover, the supernatants from BMDMs treated with the drug candidates and LPS were collected as conditioned media to evaluate their effects on VSMC proliferation and migration. Consistent with their effect on macrophage polarization, daidzein‐, mycophenolic acid‐, and artesunate‐treated conditioned medium significantly reduced the proliferation rate (Figure 6D) and migration rate (Figure 6E) of VSMCs. Notably, artesunate demonstrated the highest potency in the aforementioned assay and was subsequently selected for further evaluation.

Western blotting demonstrated that the optimal concentration of artesunate to upregulate IRF4 expression was 10 µM (Figure S6F, Supporting Information), and at this concentration, endogenous IRF4 expression in BMDMs was significantly upregulated by artesunate treatment both with and without LPS (Figure 6F; Figure S6G, Supporting Information). Further, *Irf4* and *Klf4* mRNA levels in LPS‐ or IL‐4‐ treated Lyz2‐Cre BMDMs, but not in Lyz2‐*Irf4*‐KO and Lyz2‐*Klf4*‐KO BMDMs, were significantly increased when treated with artesunate (Figure S7A, B, Supporting Information). In conjunction, the mRNA levels of *Cox‐2, Il‐6, Tnf‐α*, and *Il‐β* in LPS‐treated Lyz2‐Cre BMDMs, but not in Lyz2‐*Irf4*‐KO and Lyz2‐*Klf4*‐KO BMDMs, were decreased when treated with artesunate (Figure S7C, Supporting Information), and similar phenomena could be observed in IL‐4‐treated Lyz2‐*Irf4*‐KO and Lyz2‐*Klf4*‐KO BMDMs (Figure S7D, Supporting Information). Additionally, these results were further confirmed by Western blotting assay (Figure S8A‐D, Supporting Information). Meanwhile, when conditioned medium from artesunate‐treated BMDMs was applied to VSMCs, the proliferation and remodeling markers in VSMCs, including *Ki‐67, Pcna, Mmp2*, and *Mmp9*, were significantly decreased (Figure S8E, Supporting Information). RNA‐seq analyses also showed significantly suppressed macrophage activation and inflammation‐associated genes in the artesunate‐treated BMDMs (Figure 6G,H; Figure S9A, Supporting Information). Furthermore, artesunate treatment attenuated the activation of a broad spectrum of genes related to the proliferation and migration of conditioned medium‐treated VSMCs (Figure 6I,J). Finally, further mechanism data showed that artesunate dramatically increased the phosphorylation of signal transducer and activator of transcription 6 (pSTAT6) (Figure S9B, Supporting Information), and artesunate‐biotin was able to pull down the STAT6 protein (Figure S9C, Supporting Information). In addition, the expression of pSTAT6, IRF4, KLF4 and Arg‐1 activated by IL‐4 and artesunate were abrogated by pSTAT6 inhibitor AS1517499 (Figure S9D, Supporting Information).

### 2.7 Artesunate Attenuates Arterial Neointima Formation in Mice

**Figure 7:**
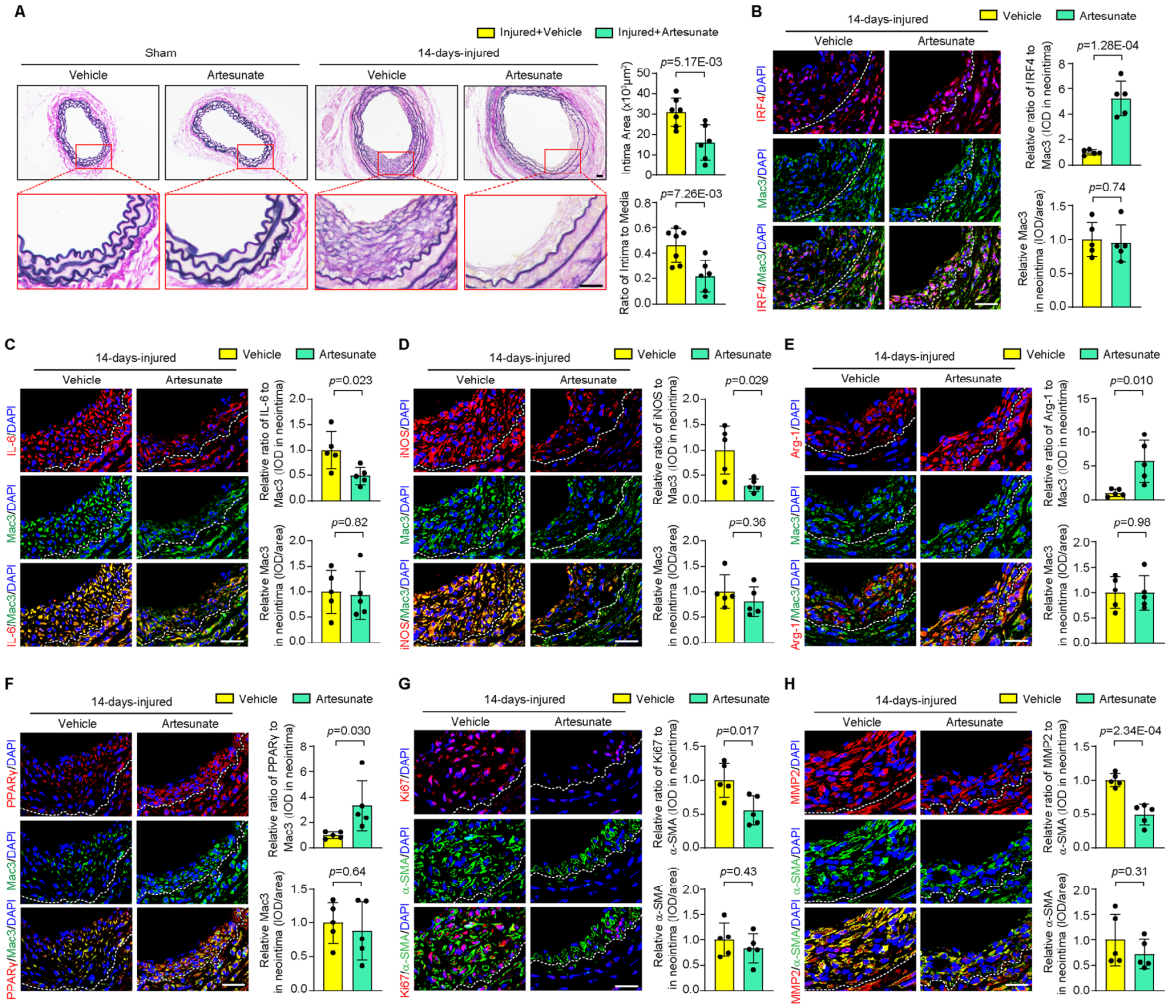
Artesunate attenuates arterial neointima formation in mice. A) EVG‐stained sections show the intimal areas in the vehicle‐ and artesunate‐treated mice after sham or at 14 days post wire‐induced carotid injury. Quantified intimal area and the intima‐to‐media (I/M) ratio. *n* = 6 to 7 per group. Scale bar = 25 µm. B–F) Immunofluorescence costaining of IRF4 (B), IL‐6 (C), iNOS (D), Arg‐1 (E), or PPARγ (F) (red) with Mac3 (green) in arteries from vehicle‐ and artesunate‐treated mice. Immunofluorescence data are graphically presented as the relative positive area of IRF4, IL‐6, iNOS, Arg‐1, or PPARγ immunostaining normalized to Mac3 and the relative expression level of Mac3 in the neointima. *n* = 5 per group. Scale bar = 25 µm. G,H) Immunofluorescence costaining of Ki67 (G) or MMP2 (H) (red) with α‐SMA (green) in arteries from vehicle‐ and artesunate‐treated mice. *n* = 5 per group. Scale bar = 25 µm. Immunofluorescence data are graphically presented as the relative positive area of Ki67 or MMP2 immunostaining normalized to α‐SMA and the relative expression level of α‐SMA in the neointima. IOD, Integrated Optical Density. Data information: The data with error bars are presented as mean ± SD. Two‐tailed Student's t‐test is used.

To examine the potential efficacy of artesunate in vivo, we evaluated the effect of artesunate on vascular restenosis in mice. Mice with carotid arterial injury were randomized into the vehicle and artesunate treatment groups. There was no obvious neointima formation in the carotid artery in sham controls (**Figure** **7**A). The neointimal area and the I/M ratio were significantly reduced in artesunate‐treated mice compared with vehicle‐treated mice 14 days after injury (Figure 7A). Immunofluorescence staining showed that, compared to the vehicle group, the expression of IRF4 in neointimal Mac3‐positive macrophages was upregulated in the artesunate‐treated mice (Figure 7B). Likewise, the expression of IL‐6 and iNOS (M1 phenotype) was downregulated, but the levels of Arg‐1 and PPARγ (M2 phenotype) were increased in the artesunate‐treated mice compared with the vehicle controls 14 days after injury (Figure 7C–F). Moreover, immunofluorescence staining for Ki67 and MMP2 in SMCs demonstrated that artesunate treatment inhibited VSMC proliferation and remodeling (Figure 7G,H). These data suggest that artesunate has a protective effect against arterial neointima formation, partly by upregulating IRF4 expression and promoting M2 macrophage polarization in mice.

### 2.8 Artesunate Attenuates Arterial Neointima Formation in Nonhuman Primates

**Figure 8:**
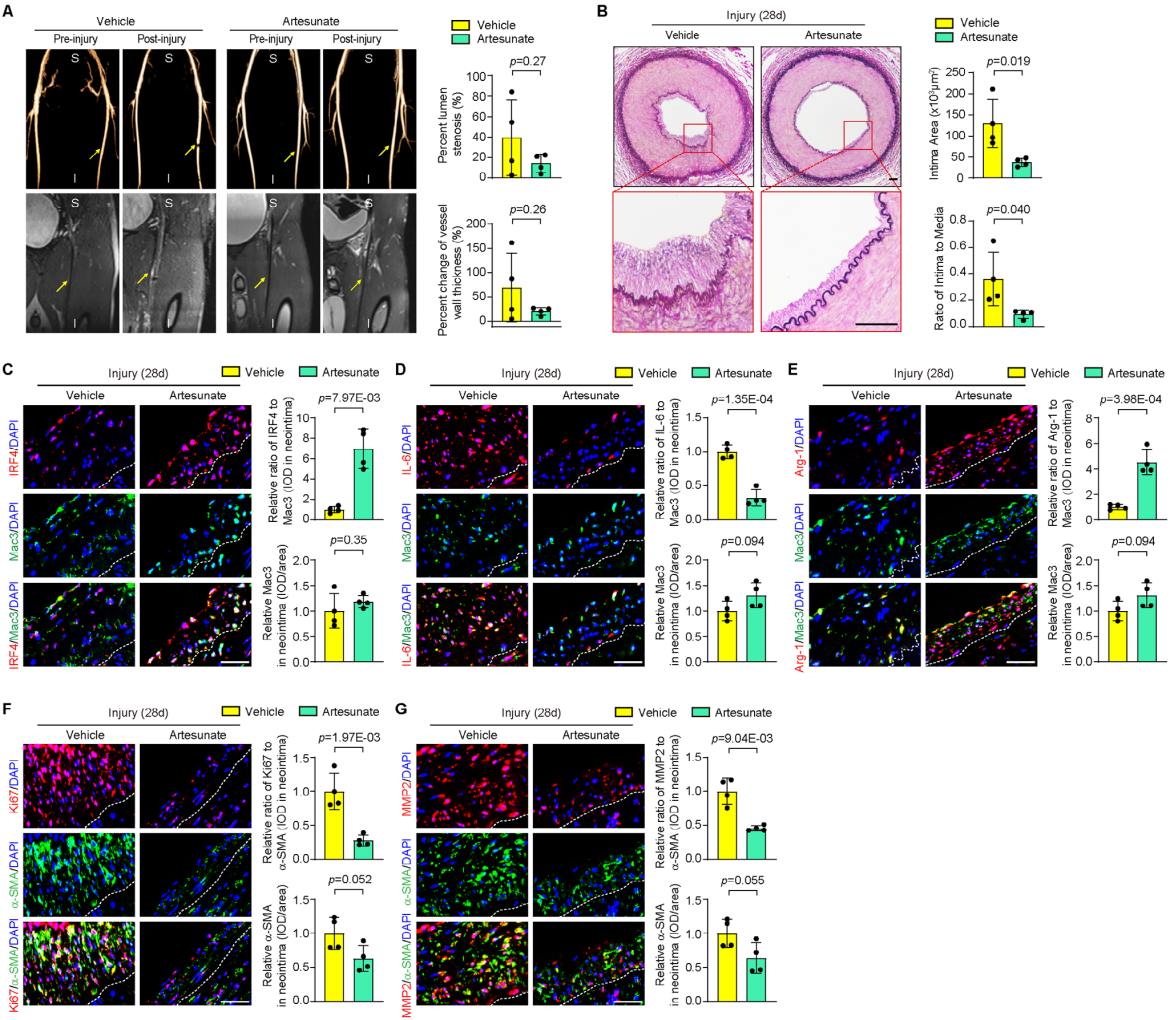
Artesunate attenuates arterial neointima formation in nonhuman primates. A) In rhesus monkey treated with vehicle and artesunate pre‐ and post‐balloon‐induced femoral artery injury, 3D volume rendering reconstruction of enhanced magnetic resonance angiography (MRA) images (upper row) showing the luminal blood flow and 3D turbo spin echo T2‐weighed (black blood) MR images (lower row) showing the vascular wall. The percent lumen stenosis and percent change in vessel wall thickness were calculated to assess neointima formation. Arrows indicate the area of arterial injury. S, superior. I, inferior. *n* = 4 per group. B) EVG‐stained sections demonstrating the intimal areas in the vehicle‐ and artesunate‐treated monkeys at 28 days post balloon‐induced femoral artery injury. Quantified intimal area and the I/M ratio. *n* = 4 per group. Scale bar = 100 µm. C–E) Immunofluorescence costaining of IRF4 (C), IL‐6 (D), or Arg‐1 (E) (red) with Mac3 (green) in arteries from vehicle‐ and artesunate‐treated monkeys. Immunofluorescence data are graphically presented as the relative positive area of IRF4, IL‐6, or Arg‐1 immunostaining normalized to Mac3 and the relative expression level of Mac3 in the neointima. *n* = 4 per group. Scale bar = 20 µm. F,G) Immunofluorescence costaining of Ki67 (F) or MMP2 (G) (red) with α‐SMA (green) in arteries from vehicle‐ and artesunate‐treated monkeys. Immunofluorescence data are graphically presented as the relative positive area of Ki67 or MMP2 immunostaining normalized to α‐SMA and the relative expression level of α‐SMA in the neointima. *n* = 4 per group. Scale bar = 20 µm. IOD, Integrated Optical Density. Data information: The data with error bars are presented as mean ± SD. Two‐tailed Student's t‐test is used.

To further explore the translational implications of artesunate treatment in arterial stenosis, we evaluated the effect of artesunate on balloon injury‐induced femoral artery neointima formation in rhesus monkeys. Balloon injury‐induced arterial stenosis 28 days after injury was measured by MRI, and the results showed that the percent lumen stenosis and percent change in arterial wall thickness in the artesunate treatment group tended to decrease (**Figure** **8**A). This protective effect on vascular restenosis was substantiated by histological analysis based on the reduced intima area and I/M ratio (Figure 8B). In line with the observations in mouse models, artesunate treatment significantly augmented IRF4 expression in the injured arteries compared with vehicle controls (Figure 8C), along with the decreased expression of IL‐6 (Figure 8D) and the increased expression of Arg‐1 (Figure 8E) in neointimal Mac3‐positive macrophages. Expression of Ki67 and MMP2 in neointimal α‐SMA‐positive SMCs were also significantly downregulated in the injured arteries from the artesunate‐treated monkeys, compared to the vehicle controls (Figure 8F,G). Therefore, these data support that artesunate ameliorates arterial stenosis in nonhuman primates.

## 3 Discussion

Vascular restenosis and the need for repeated revascularization remain a prevalent clinical challenge globally.^[^ ^5^, ^25^ ^]^ Through an integrated analysis of post‐injury vasculature in mice and nonhuman primates, we have discovered that macrophage IRF4 serves as a protective effector against arterial restenosis. This effect is mediated, in part, by IRF4‐induced KLF4 expression and the subsequent modulation of M2 macrophage polarization. Furthermore, a high throughput drug screening based on IRF4 transcriptional induction identified an FDA‐approved drug, artesunate, as a potent activator of IRF4. Its therapeutic effect against vascular injury‐induced restenosis was demonstrated in vivo across different species. Thus, our study uncovers a previously unappreciated role of IRF4 in vascular protection by modulating inflammatory responses and highlights a potential therapeutic approach against injury‐induced remodeling.

Arterial injuries resulting from intravascular stenting, endarterectomy, and angioplasty trigger complex and dynamic inflammatory responses,^[^ ^26^ ^]^ where infiltrating monocytes and monocyte‐derived macrophages have pivotal impact on the entire process from inflammation to repair in post‐injury remodeling.^[^ ^27^ ^]^ In the first week after stenting, nearly 40% of neointimal cells are infiltrated macrophages.^[^ ^28^ ^]^ As established in the literature, macrophages are highly heterogeneous at the functional and molecular levels in the post‐injury vasculature, and are generally characterized by their M1 versus M2 polarization status.^[^ ^13^ ^]^ M1 macrophages secrete proinflammatory factors and promote VSMC proliferation and migration, while M2 macrophages exhibit anti‐inflammatory properties and reduce the incidence of in‐stent restenosis.^[^ ^13^, ^14^ ^]^ However, the molecular mechanisms for macrophage polarization regulation, particularly in vascular restenosis, remain to be fully elucidated. The therapeutic potential of targeting macrophage polarization to treat this major disease has not yet been clearly demonstrated.

Among the myriads of molecular pathways controlling macrophage polarization, a number of transcription factors have been uncovered that play pivotal roles in modulating macrophage phenotypes.^[^ ^15^, ^16^ ^]^ IRFs are hub transcription factors that were initially discovered to regulate immune responses during viral infections.^[^ ^17^, ^18^ ^]^ Earlier studies from our group also found that IRF4 is expressed and exerts its effects in nonimmune cells, which is further confirmed in this study, where several other cell types, in addition to macrophages, in the vascular neointima also express IRF4 (Mac3^−^IRF4^+^ cells). As previously reported, IRF4 promotes cardiac hypertrophy in cardiomyocytes^[^ ^29^ ^]^ and has neuroprotective effects during stroke.^[^ ^30^ ^]^ Through VSMC‐specific knockout and transgenic over‐expression in mice, our group has revealed that IRF4 inhibits VSMC proliferation and migration, thus having a protective function against restenosis in post‐injury arteries.^[^ ^23^ ^]^ In addition, IRF4 has been implicated as an anti‐inflammatory mediator in dendritic cells^[^ ^31^ ^]^ and macrophages.^[^ ^32^, ^33^ ^]^ However, the effects of IRF4 on macrophage polarization and subsequent neointima hyperplasia, particularly in drug screening targeting IRF4 and efficacy evaluation, still require extensive study. Consistent with previous reports,^[^ ^19^, ^20^ ^]^ we found that macrophage IRF4 skewed the M1/M2 polarization balance toward the anti‐inflammatory M2 type in vitro and in vivo during post‐injury vascular remodeling. Further molecular studies uncovered KLF4 as a key downstream factor in IRF4‐mediated regulation of macrophage polarization. This observation aligns with the previously reported role of KLF4 in the monocyte differentiation^[^ ^34^, ^35^ ^]^ and vascular pathology.^[^ ^36^ ^]^ Since macrophage infiltration is an early event in the inflammatory cascade that leads to VSMC proliferation/migration and neointima formation after endothelial injury,^[^ ^37^, ^38^ ^]^ IRF4‐mediated macrophage polarization should provide early protection against neointimal formation. In our current work, this concept was validated in vivo through macrophage‐specific manipulation of IRF4 expression both in mice and nonhuman primates, further supporting its potential for clinical translation.

Given the pivotal role of macrophages in neointima formation, systemic inactivation or depletion of macrophages has been proposed as a therapeutic strategy.^[^ ^39^ ^]^ However, such blunt treatment may have unintended adverse consequences, such as fatal infection due to non‐specific immunosuppression.^[^ ^40^ ^]^ Furthermore, due to the complexity of macrophage‐mediated signaling pathways in CVD pathogenesis, the mere applications of cell mediators (such as IL‐1, IL‐10, and MMP‐9) often fail to yield expected benefits, imposing the challenges encountered in most cardiovascular clinical trials.^[^ ^41^, ^42^ ^]^ Therefore, it is imperative to investigate mechanism‐based macrophage phenotype regulatory molecules that are integral to the pathological vascular remodeling, and to ensure that these targets do not trigger serious adverse effects. In this study, we discovered an unexpected effect of artesunate as a potent activator of IRF4 expression in macrophages through unbiased high‐throughput screening using a library of FDA‐approved compounds. Artesunate, a semi‐synthetic derivative of artemisinin, is an effective first‐line antimalarial agent recommended by the World Health Organization (WHO) for severe malaria, and has a worldwide safety record for adults, children and pregnant women.^[^ ^43^, ^44^ ^]^ Additionally, artesunate has been reported to have therapeutic properties against infections, cancers, autoimmune diseases, and atherosclerosis.^[^ ^45^, ^46^, ^47^ ^]^ Early reports have shown that artemisinin ameliorates inflammatory bowel disease by promoting M2 polarization^[^ ^48^ ^]^ and alleviates atherosclerotic lesions by promoting macrophage autophagy.^[^ ^48^ ^]^ Our screening results indicate that artesunate enhances IRF4 expression by modulating the IRF4 promoter activity. To further explore the interaction between artesunate and IRF4, as well as its impact on M2 polarization, we initially analyzed the potential signals linked to artesunate and macrophage polarization. Previous studies have reported that the STAT6 signaling pathway is crucial for IRF4 expression and participates in M2 activation.^[^ ^50^ ^]^ M2 polarization occurs via the STAT6‐IRF4 signaling pathway.^[^ ^51^, ^52^ ^]^ Moreover, artesunate has been shown to enhance pSTAT6 expression in macrophages, suggesting that STAT6 is a key regulator in balancing M1/M2 polarization and mediating the anti‐colitis effects of artesunate.^[^ ^53^ ^]^ Similarly, our results demonstrate that IRF4 and pSTAT6 induction is pivotal to artesunate‐mediated anti‐inflammatory function and artesunate is proposed as a new anti‐restenosis therapy in both rodent and nonhuman primates. Further, since macrophage‐rich areas are prevalent in the lesion of inflammatory vascular diseases, such as atherosclerosis,^[^ ^54^ ^]^ graft vasculopathy,^[^ ^55^ ^]^ and autoimmune diseases associated vasculitis,^[^ ^56^ ^]^ it is worthwhile to investigate the therapeutic potential of artesunate in these vascular disorders.

The recent clinical trials showed that anti‐inflammatory strategies have successfully reduced cardiovascular events, which give rise inflammation as a rational target for treating CVD. Canakinumab, a neutralizing antibody against IL‐1β (mainly produced by macrophages), is the first drug shown in clinical trials to specifically constrain inflammation and the recurrence rate of cardiovascular events.^[^ ^7^ ^]^ Colchicine, a broad anti‐inflammatory drug, significantly reduces the risk of cardiovascular event in patients with myocardial infarction and chronic coronary artery disease.^[^ ^8^, ^10^ ^]^  Furthermore, the most common autoimmune rheumatic diseases, such as rheumatoid arthritis and systemic lupus erythematosus, are associated with increased adverse cardiovascular risk, which further proves that inflammatory process per se is the risk factor for CVD and related complications.^[^ ^3^, ^57^, ^58^ ^]^ Given the conclusive relationship between autoimmune diseases and cardiovascular events, more anti‐inflammatory drugs for rheumatism are being tested in clinical trials for CVD. For instance, hydroxychloroquine and methotrexate have both been found to reduce CVD risk in rheumatoid arthritis.^[^ ^59^, ^60^ ^]^ Sarilumab and ziltivekimab, monoclonal antibodies targeting the IL‐6 receptor and IL‐6, are being evaluated for their effects on cardiovascular risk in patients with rheumatoid arthritis (NCT04350216) and chronic kidney disease (NCT05021835) with high CRP levels, respectively. Etanercept, a TNF‐α blocker, is also undergoing clinical trials in patients with acute myocardial infarction (NCT01372930). In our work, due to its anti‐inflammatory activities, artesunate represents a promising cardiovascular medicine, and its demonstrated efficacy further supports the potent role of inflammation in restenosis. Over time, we anticipate that, in addition to lipid‐lowering therapy, combination therapies with anti‐inflammatory agents will become the standard of care for patients with CVD, especially restenosis.

There are still some limitations in the current study. While cell culture, mouse and monkey experiments provide essential and highly valuable approaches to exploring mechanisms and therapeutic strategies, a comprehensive analysis of the IRF4‐KLF4 effects on macrophage polarization and vascular restenosis in clinical cases is needed in the future. Second, only male animal models were performed in our study to avoid the unknown impact of periodic changes in the abundance of estrogen in female animals throughout the menstrual cycle. This limitation leads to insufficient exploration of the benefits and potential side effects of artesunate for clinical translation in females. Further, the therapeutic efficacy of artesunate in reversing disease progression, off‐target effects, timing and dosage of administration, and potential side effects from long‐term treatment remains to be studied. However, drug delivery using the drug‐eluting stent allows local enrichment of artesunate to a vascular segment to reduce neointimal hyperplasia without causing serious systemic side effects is one of the possible strategies.

## 4 Conclusion 

Collectively, we revealed the novel role of IRF4 in macrophage polarization with a potent protective effect against arterial injury‐induced restenosis. This beneficial effect is mediated, in part, by IRF4‐dependent induction of KLF4 expression and subsequent M2 macrophage polarization. In particular, artesunate was screened and identified to possess unexpected anti‐inflammatory activity, and exhibited conserved therapeutic efficacy for vascular restenosis in rodents and primates. Overall, this study reveals a new mechanistic basis and a novel therapeutic approach for vascular restenosis and related inflammatory cardiovascular diseases.

## 5 Experimental Section

### Study Design

The primary goal of this study was to explore the key determinant of inflammation‐derived vascular restenosis. To this end, transcriptome‐wide cross‐species gene expression analysis was performed and identified IRF4 as a promising driver of macrophage polarization and vascular restenosis. Then, myeloid‐specific and targeted gain‐ and loss‐of‐function of IRF4 models were established and characterized on injury‐induced artery neointima formation and macrophage polarization in mice or nonhuman primates. The molecular mechanisms underlying the anti‐inflammatory effects of IRF4 were determined by bioinformatics analysis and ChIP assay, followed by biological exploration and validation. Finally, small molecule screening for IRF4 transcriptional activators was implemented based on a reporter assay and the top candidate was validated in mouse and nonhuman primate models of arterial restenosis. The sample size of animals was determined based on past experience and published literature and was specified in the figure legends. Animals with identical background were randomly assigned to each group. Histopathological evaluation was performed in a double‐blind manner. In vitro, experiments were carried out at least three replicates unless otherwise specified.

### Experimental Animals

All animal protocols were approved by the Animal Care and Use Committee of the National Translational Science Center for Molecular Medicine (approval number: 2020‐NTSCMM‐ID005) and Zhongnan Hospital of Wuhan University (approval number: ZN2022116). Animal experiments were conducted according to institutional ethical guidelines. The animals received humane care according to the Guide for the Care and Use of Laboratory Animals published by the National Academy of Sciences and the National Institutes of Health. Male mice aged 10 to 12 weeks and weighing 20 to 30 g were included in the experiments. Mice were housed strictly inbred in pathogen‐free individually ventilated cages (3‐5 mice per cage) with free access to clean tap water and rodent chow. For monkey experiments, male rhesus monkeys aged 5 to 7 years (5 to 12 kg) were purchased from Topgene Biotechnology (Guangzhou, China). The monkeys included in this study were healthy, passed the physical examination, and met the standards of local quarantine inspection. The monkdeys were individually housed and acclimated for at least 1 week before experiments. All monkeys were fed a regular chow diet twice daily, seasonal fruits once daily, and clean water freely. The mice and monkeys were housed at a controlled temperature (23 ± 2 °C), humidity (50 to 65%) and photoperiod [12:12 hour light/dark cycle].

### Vascular Injury Models in Mice

Wire injuries induced neointima formation in the mouse carotid artery, as previously described.^[^ ^61^ ^]^ Briefly, after anesthetization (sodium pentobarbital, 80 mg kg^−1^, intraperitoneally), the left carotid artery was carefully exposed via a midline neck incision. The left external carotid artery was ligated proximally to the bifurcation point with 8‐0 silk suture (Ethicon). Additional vascular clamps temporarily interrupted the internal and common carotid arterial blood flow. A transverse arteriotomy was made in the external carotid artery, and a flexible angioplasty guidewire was introduced into and withdrawn from the arterial lumen five times with a rotating motion. Then, the wire was removed, and vascular clamps were released to resume blood flow. The skin incision was closed with a single suture. For treatment experiments, the mice received either a vehicle and artesunate following sham or vascular injury for 14 days. Since the maximum oral dose and rectal dose of artesunate for clinical application in malaria treatment is 8 mg kg^−1[^ ^62^ ^]^ and 10 mg kg^−1^,^[^ ^63^ ^]^ respectively, according to the animal equivalent dose (AED) calculation,^[^ ^64^ ^]^ the AED for mice is approximately 98.4‐123 mg kg^−1^. Additionally, considering the reported doses from previous studies on artesunate administration in mice, the dosage of 100 mg kg^−1^ (gavage, qd, Sigma‐Aldrich) was ultimately used for mice in this study. At 14 days post‐injury, mice were euthanized via an overdose of sodium pentobarbital (150 mg kg^−1^). Both injured left and uninjured right carotid arteries were excised.

### Vascular Injury Models in Rhesus Monkeys

For the monkey model of intimal hyperplasia, injury to the femoral artery was achieved as described below. Monkeys were first concurrently anesthetized with atropine (0.04 mg kg^−1^), xylazine (0.1 mL kg^−1^), and Zoletil (15 mg kg^−1^) by intramuscular injection. Once adequately anesthetized, the monkey was fixed, and their heart rate, blood pressure, and oxygen saturation were measured throughout the process. The left femoral artery and its branches were surgically exposed and carefully dissected. Then, the branches of the femoral artery were ligated from the bifurcation with 8‐0 silk sutures and the proximal and distal blood vessels were clamped. After the femoral artery puncture, the angioplasty balloon was inflated to a pressure of 12 atmospheres. The injury paradigm used three balloon inflations for 3 min each, with 1‐minute reperfusion between each inflation. After removing the catheter, the injured vessel was examined visually to ensure that blood flow was restored. The femoral artery was then surgically stapled, and the incision was sutured. After the surgery, monkeys received aspirin (25 mg kg^−1^, qd) and clopidogrel (75 mg kg^−1^, qd) in the diet for 7 days to decrease platelet activation and to prevent possible thrombosis. The monkeys were allowed to recover in their original cage. For treatment experiments, the monkeys were given vehicle or artesunate with diet following vascular injury for 28 days. The dosage of artesunate for monkeys was converted from that of the mice based on body surface area (Meeh‐Rubner formula). Considering that the average weight of mice is 25 g and that of monkeys is 10 kg, their respective body surface areas were calculated, and the dosage of artesunate for the monkeys was determined to be 17.6 mg kg^−1^ (qd, Sigma‐Aldrich). At 28 days post‐injury, monkeys were anesthetized, and the injured femoral arteries were excised.

### Genetic Modification of Mice

Myeloid‐specific genetic modification mice were generated by constructing modification elements downstream of the Lyz2 promoter.^[^ ^65^ ^]^ Lyz2‐*Irf4*‐KO (*Irf4^flox/flox^*/Lyz2‐Cre) mice were generated by mating *Irf4^flox/flox^* (stock number: 0 09380, Jackson Laboratory) mice with Lyz2‐Cre mice (stock number: 0 04781, Jackson Laboratory). To obtain myeloid‐specific IRF4 transgenic mice, full‐length mouse IRF4 cDNA was subcloned downstream of the mouse Lyz2 promoter (Lyz2‐*Irf4*‐TG mice). *Klf4‐*floxed mice were generated using the CRISPR/Cas9 system.^[^ ^23^ ^]^ Lyz2‐*Klf4*‐KO mice were generated by mating *Klf4^flox/flox^* mice with Lyz2‐Cre mice. Lyz2‐*Klf4*‐TG mice were generated by mating *CAG‐loxP‐CAT‐loxP‐Klf4* mice with Lyz2‐Cre mice. Lyz2‐*Irf4*‐KO/*Klf4*‐TG mice were generated by mating *Irf4^flox/flox^*/Lyz2‐Cre mice with *Klf4* conditional transgenic mice. Lyz2‐*Klf4*‐KO/*Irf4*‐TG mice were generated by mating Lyz2‐*Klf4*‐KO mice with *Irf4*‐TG mice. The constructed expression vectors were microinjected into fertilized C57BL/6J embryos.

### Human Samples

Human samples of coronary arteries with in‐stent restenosis were obtained from coronary heart disease (CHD) patients who previously had percutaneous coronary intervention (PCI) and were undergoing heart transplants. Coronary arteries from matched donors who wanted heart transplantation but were unsuitable for transplantation were used as control samples. The demographic and clinical characteristics of human donors are summarized in Table S1 (Supporting Information). All procedures involving human samples adhered to the principles of the Declaration of Helsinki and were approved by the ethics committees of the Renmin Hospital of Wuhan University (approval number: 2014‐K‐016). Written informed consent was obtained from each participating subjects.

### Cell Culture and Preparation of Conditioned Medium

BMDMs were isolated and differentiated from mice as previously described.^[^ ^66^ ^]^ Briefly, bone marrow was harvested from the femur and tibia of the indicated mice and filtered through a 70 µm strainer. After treatment with Red Cell Lysis Buffer (Sigma), the isolated cells were cultured in RPMI medium (Gibco) containing 10% FBS (Gibco) and 1% penicillin‐streptomycin (Gibco), and stimulated with 50 ng mL^−1^ recombinant mouse M‐CSF (Biolegend) for 5 days. BMDM purity was assessed by flow cytometry following immunostaining with anti‐CD11b and anti‐F4/80 (both from Biolegend), resulting in > 98% CD11b^+^ F4/80^+^ cells, indicating a highly pure population of BMDMs. To induce an M1‐like phenotype, BMDMs were treated with LPS (100 ng mL^−1^, Sigma) for 24 h. To obtain an M2‐like phenotype, BMDMs were stimulated with IL‐4 (10 ng mL^−1^, Biolegend) for 24 h. In some experiments, as indicated, BMDMs were treated with vehicle (DMSO), chemotherapy compounds (10 µM, Selleck Chemicals), or APTO‐253 (5 µM, MedChemExpress). To obtain a conditioned medium, BMDMs were initially stimulated with either LPS or IL‐4 for 24 h. After this stimulation period, the supernatant was removed and the cells were washed. Then, BMDMs were treated with fresh medium and incubated for an additional 24 h. Following this incubation, the supernatant was collected as conditioned medium.

The VSMC cell line generated from mouse aortic vascular smooth muscle cells (MOVAS) was obtained from the American Type Culture Collection (ATCC). The HEK 293T cell line was purchased from the Cell Bank of the Type Culture Collection of the Chinese Academy of Sciences (Shanghai, China). These cells were cultured in DMEM (Gibco) containing 10% FBS (Gibco) and 1% penicillin‐streptomycin (Gibco) at 37 °C in a 5% CO_2_‐humidified atmosphere.

### Adenovirus Construction and Injection

To generate myeloid‐specific gene expression vectors in rhesus monkeys, a CD68 gene promoter was used to construct adenovirus. The adenovirus plasmid carrying the full‐length IRF4 coding sequence (Ad‐*P*_CD68_‐IRF4) and the adenovirus GFP control plasmids (Ad‐*P*_CD68_‐GFP) were constructed by Hanbio (Shanghai, China). As described previously,^[^ ^67^ ^]^ Ad‐*P*_CD68_‐GFP and Ad‐*P*_CD68_‐IRF4 were injected intravascularly (1 × 10^9^ PFU per monkey) at 1 week after femoral artery injury. At 28 days post‐injury, monkeys were anesthetized and the femoral arteries were excised.

### Elastica Van Gieson and Immunofluorescence Staining

Carotid and femoral arteries were harvested at the indicated times post‐injury. The arterial segments were excised after circulation perfusion and fixed with 4% paraformaldehyde in PBS before dehydration in ethanol and xylene, and embedding in paraffin for further preparation for histological analysis and immunofluorescence. Each paraffin section was 5 µm in thickness.

To evaluate vascular tissue structures and the extent of carotid artery luminal occlusion, consecutive sections from the bifurcation site of the carotid artery were stained with Elastica van Gieson (EVG). Measurements included luminal area, internal elastic lamina and external elastic lamina. The intimal area and intima‐to‐media (I/M) ratio were calculated to assess neointima formation. All sections were analyzed by a single observer blinded to the study design using Image‐Pro Plus 6.0.

For immunofluorescence staining, the arterial sections were collected, placed onto gelatin‐coated glass slides, and blocked with 20% donkey serum for 1 h. Tissue sections were then incubated with corresponding primary antibodies (Table S2, Supporting Information) overnight at 4 °C, followed by incubation for 1 h with appropriate secondary antibodies. The secondary antibodies used: included Alexa Fluor 488‐conjugated donkey anti‐rat IgG (A21208; 1:200; Invitrogen, Carlsbad, CA, USA), Alexa Fluor 488‐conjugated donkey anti‐mouse IgG (A21202; 1:200; Invitrogen), Alexa Fluor 568‐conjugated donkey anti‐rabbit IgG (A11042; 1:200; Invitrogen) or Alexa Fluor 568‐conjugated donkey anti‐mouse IgG (A10037; 1:200; Invitrogen). After nuclear staining for 30 s with 4′,6‐diamidino‐2‐phenylindole (DAPI), the slides were imaged using an inverted confocal microscope (TCS SP8, Leica, Germany).

### Multiplex Immunohistochemistry Assay

Briefly, the tissue sections were dewaxed, followed by antigen retrieval in TRIS‐EDTA (pH = 9) buffer. After blocked with 5% goat serum in PBS, the tissue sections were sequentially incubated with primary antibodies. After being washed, the bound antibodies were reacted with goat anti‐rabbit IgG (Panovue, Beijing, China) and stained using the tyramine signal amplification (TSA)‐Indirect kit (PerkinElmer, USA), according to the manufacturer's manual. The primary and secondary antibodies were removed by microwaving, and then blocked again by blocking solution. The sections were stained with another primary antibody, and the above processes were repeated. Finally, DAPI was used to stain nuclei. Images were analyzed with HALO Image analysis software and Inform software (version 3.0).

### Magnetic Resonance Imaging (MRI) and Analysis

The vehicle‐ and artesunate‐treated monkeys were imaged by high‐resolution MRI with a 3.0‐Tesla (Magnetom Prisma, Siemens Healthcare GmbH, Germany) before and 28 days after balloon‐induced femoral artery injury. The MRI protocol consisted of the following 4 steps: (1) gradient echo series to localize anatomic structures; (2) time‐of‐flight (TOF) magnetic resonance angiography (MRA) to localize the vascular stenosis and provide anatomic landmarks to define the precise location of the stenosis; (3) unenhanced T2‐weighted 3D isotropic TSE sequences to show the vascular wall; and (4) pre‐ and post‐contrast fast low‐angle shot 3D (fl3d) MRA sequences to show blood flow in the femoral artery. Contrast‐enhanced MRA was performed with 2.5 mL s^−1^ of 0.1 mL kg^−1^ gadobutrol (Gadovist; Bayer Vital GmBH, Leverkusen, Germany). The distance between the common femoral artery bifurcation and maximum stenosis was used to predefine anatomic landmarks and obtain reproducible image positions. Subsequent angiography sequences were performed using this superficial receive‐only coil centered at the stenosis level. A blinded operator performed computer‐assisted morphometric analysis of MRI images, including measuring vascular lumen diameter and wall thickness by manually tracing vascular borders (RadiAnt DICOM Viewer, Medixant, Poznan, Poland). Percent lumen stenosis was calculated according to the following formula: 100 × [(diameter of stenosis distal lumen) − (diameter of the lumen at stenosis) / (diameter of stenosis distal lumen)]. The percent change in vessel wall thickness was calculated as follows: 100 × [(diameter of the vessel wall at stenosis) − (diameter of stenosis distal vessel wall) / (diameter of the vessel wall at stenosis)]. Vascular landmarks were used to match the MR images.

### EdU Incorporation Assay

VSMCs were cultured in a 96‐well microplate overnight, followed by serum starvation for 24 hours. Then starved VSMCs were treated with conditioned medium. After 24 h, VSMC proliferation was analyzed via a BeyoClick EdU Cell Proliferation Kit with Alexa Fluor 555 (Beyotime, China) according to the manufacturer's instructions. Briefly, cells were incubated with EdU (10 µM) for 2 h at 37 °C and subsequently fixed with 4% paraformaldehyde. Then, the cells were incubated with the permeable solution and the Click Reaction Solution containing Azide 555 at room temperature. Cell nuclei were stained with Hoechst 33 342 dye. Images of the stained cells were taken and recorded by an Operetta CLS High Content Analysis System (Perkin Elmer).

### BrdU Incorporation Assay

VSMCs were cultured overnight in 96‐well microplate and subsequently serum‐starved for 24 hours. The starved VSMCs were treated with conditioned medium from BMDMs. After 24 h, VSMCs proliferation was assessed using a BrdU cell proliferation kit (Roche, Basel, Switzerland) following the manufacturer's instructions. Briefly, the cells were incubated with BrdU for 2 h, then fixed, and their DNA denatured and labeled with a peroxidase‐conjugated BrdU antibody. After incubation with the peroxidase substrate and acid stop solution, absorbance values representing cell proliferation were measured by a microplate reader at 450 nm (OD450) with reference measurement at 490 nm (OD490).

### Migration Assay

The migration of VSMCs was evaluated via the Transwell chamber assay. After serum starvation for 24 h, VSMCs (1 × 10^5^ per well) were added to the upper chamber (8 µm pore size, Corning) in 200 µL of serum‐free medium. The lower chamber was filled with cultured BMDMs stimulated with LPS (100 ng mL^−1^) or IL‐4 (10 ng mL^−1^). After 10 h of incubation, cells were fixed with 4% paraformaldehyde for 20 min and stained with 0.1% crystal violet for 10 min. The non‐migrating VSMCs on the upper chamber of the filter were removed with a cotton bud. The cells on the underside were imaged and counted under a phase contrast microscope for quantification.

### Quantitative Real‐Time PCR

Total RNA was extracted from cells and tissues via TRIzol reagent (T9424, Sigma‐Aldrich) and reverse‐transcribed into complementary DNA by Transcriptor HiScript III RT SuperMix for qPCR (+gDNA wiper) (Vazyme). Then, SYBR Green PCR Master Mix (0 488 735 2001, Roche) and a LightCycler 480 Real‐time PCR System (Roche) were used to quantify the mRNA levels of the target genes in accordance with the manufacturer's protocol. The relative expression levels of the target genes were calculated and normalized to GAPDH mRNA expression. The PCR primer pairs used in this study are listed in Table S3 (Supporting Information).

### Western Blotting

Total proteins from tissue or cell samples were lysed using RIPA assay buffer (65 mM Tris‐HCl pH 7.5, 150 mM NaCl, 1 mM EDTA, 1% NP‐40, 0.5% sodium deoxycholate, 0.1% SDS, 13 protease inhibitor cocktail and 13 phosphatase inhibitor PhosStop) and quantified with a BCA Protein Assay Kit (Thermo Fisher Scientific). Protein samples were separated using 10% SDS–PAGE gels and then transferred to polyvinylidene difluoride (PVDF) membranes (Millipore). After blocking with 5% skim milk for 1 h at room temperature, the membranes were incubated with the appropriate primary antibodies (Table S2, Supporting Information) overnight at 4 °C. Then, the membrane was incubated with secondary horseradish peroxidase (HRP)‐conjugated antibodies for 1 h at room temperature and visualized via a FluorChem E imager (ProteinSimple, FluorChem E). GAPDH was used as the loading control.

### Pull‐Down Assay

BMDMs from mice were incubated with biotin, artesunate, and artesunate‐biotin probes for 2 h, respectively. The cells were lysed using RIPA lysis buffer. After centrifugation at 12 000 rpm for 15 mins, the supernatant was incubated with BeaverBeads Streptavidin (Beaver, China) on a rotary mixer at room temperature for 1 h. The mixture was then separated using a magnetic separator, and the supernatant was transferred to a new centrifuge tube. Subsequently, 1 mL of PBS (containing 0.05% Tween‐20) was added, followed by magnetic separation and removal of the supernatant. The washing process was repeated five times, and 100 µL of 1 × SDS‐PAGE Loading Buffer was added to elute the sample proteins. The expression levels of the collected proteins were detected by Western blotting assay.

### Chromatin Immunoprecipitation (ChIP)

ChIP was performed via a ChIP Assay Kit (P2078, Beyotime) according to the manufacturer's instructions. Briefly, macrophages were stimulated with LPS for 4 h, followed by fixation with 1% formaldehyde. After washing with ice‐cold PBS (1 mM PMSF), cells were resuspended in SDS lysis buffer. Then cell lysates were sonicated at low power (28 cycles) by a Bioruptor Sonication Systemat. The samples were precleared with Protein A/G Agarose and then incubated with goat‐anti‐IRF4 (sc‐6059, 1 mg mL^−1^, goat, Santa Cruz Biotechnology) or normal goat IgG (sc2028, 0.2 mg mL^−1^, goat, Santa Cruz Biotechnology) overnight. After incubation with Protein A/G Agarose for 2 h, the antibody/protein/DNA complexes were washed with a series of wash buffers. The DNA‐protein complexes and input control were eluted with elution buffer (1% SDS and 0.1 M NaHCO_3_) and de‐crosslinked by 0.2 M NaCl incubation. Then, the samples were treated with proteinase K, and the DNA fragments were purified using a FastPure Gel DNA Extraction Mini Kit (Vazyme). mIL12‐pro84 was used as a positive control, and P6 was used as a negative control. The primers for ChIP‐PCR were as follows: P1, 5‐CTTTCTTTCTTGACACCACAATGGG‐3 (forward) and 5‐AGGTGTTCTTTTTGTTGTTCCTTTG‐3 (reverse); P2, 5‐CTTTGACAGACTGACTGGGC‐3 (forward) and 5‐ATAGGTCCCAAGGATTCCG‐3 (reverse); P3, 5‐TCTGTGGTCGGCCAGAAGT‐3 (forward) and 5‐GTGAAACGACAGAGGACGTGG‐3 (reverse); P4, 5‐TCACCTCCCAGTGAAGTCCC‐3 (forward) and 5‐GACCTTTACCTCGCCGCTTC‐3 (reverse); P5, 5‐ATGACTACGTGCCCAATCCC‐3 (forward) and 5‐CGGAACCCCTTCTCCTAGCTT‐3 (reverse); P6, 5‐TGGCGGACATCAATGACGTG‐3 (forward) and 5‐CTGAAGGCGTCTCTGACCAC‐3 (reverse).

### Dual‐Luciferase Reporter Assay

Screening of the potential compounds that could affect IRF4 expression was performed by dual‐luciferase reporter assay. A total of 3000+ compounds from the FDA‐approved Drug Library (L1300, Selleck Chemicals) were used for high‐throughput screening. HEK 293T cells were seeded in a 10‐cm Petri dish (5 × 10^6^ per dish) and co‐transfected with a firefly luciferase reporter plasmid encoding the IRF4 promoter (4 µg) and a Renilla luciferase reporter plasmid (4 µg). After 24 h of transfection, the cells were seeded in 96‐well plates (8 × 10^4^ per well) and an individual library compound (10 µM) was added to each well. Then the cells were lysed with Passive Lysis Buffer (Promega) after 12 h of incubation. The supernatant was then collected from the cellar lysate for the luciferase activity assay. The firefly and Renilla luciferase activities were measured by a Dual‐Luciferase Reporter Assay System (Promega) according to the manufacturer's instructions. Firefly luciferase activity was normalized to the activity of Renilla for each sample. The inhibitory ability of the screening compounds was presented and ranked by log2‐fold change normalized to the control. All experiments were performed in triplicate. Finally, the corresponding compounds that inhibited *IRF4* luciferase reporter activity more than twofold are shown in Figure 6A.

### Luciferase Reporter Assays

By amplifying the IRF4 gene‐coding region with the primers 5‐CGGAATTCATGAACTTGGAGACGGGCAGC‐3 (forward) and 5‐CCGCTCGAGTCACTCTTGGATGGAAGAAT‐3 (reverse) from the cDNA library, the plasmid psi‐flag‐IRF4 was generated after cloning the amplicon into the psi‐flag plasmid between the EcoRI and XhoI sites. The luciferase reporter plasmid pGL3‐pmKLF4 was generated by amplification of the KLF4 promoter from the C57BL/6 genome (NC_000076.6) with the primers 5‐CTAGTCTAGATATGCTGGAGCATCGACCC‐3 (forward) and 5‐GGAAGATCTCGCGCTACGATCACTCCG‐3 (reverse) and ligation into the pGL3‐basic vector (Promega, Madison) between the NheI and BglII sites. The potential IRF4 binding sites in the KLF4 promoter were predicted using Genomatix (http://www.genomatix.de/) online software. The ISRE core sequence deletion plasmid pGL3‐pmKFL4‐del was generated using the fusion‐PCR approach described earlier with the primers 5‐CCTCTTCCCAGCGCAGGAACCTCTTTATGACTCACTTCTC‐3 and 5‐GAGAAGTGAGTCATAAAGAGGTTCCTGCGCTGGGAAGAGG‐3. Primary macrophages were co‐transfected with 2 µg of psi‐flag‐IRF4, 100 ng of pGL3‐pmKFL4 or pGL3‐pmKFL4‐del and 1 ng of pRL‐TK‐Luc for 24 h. The cells were treated with LPS for 4 h and then harvested and lysed with 100 µL of passive lysis buffer (Promega). After removing the cell debris by centrifugation, the supernatant was analyzed using a Single‐Mode SpectraMax Microplate Reader in accordance with the manufacturer's instructions.

### RNA‐Seq Data Analysis

For the RNA‐Seq assay, total RNA was isolated by TRI Reagent (T9424, Sigma‐Aldrich). The quality of the extracted total RNA was assessed using an RNA 6000 Nano kit (5067‐1511, Agilent, CA, USA). RNA‐seq libraries were prepared by an MGIEasy RNA Library Prep Kit (1 000 006 384, MGI Tech Co., Ltd, Shenzhen, China) according to the manufacturer's instructions. For gene expression analysis, samples were sequenced on a BGISEQ‐500 (MGI Tech Co.) with a single‐end 50 bp module. The clean reads were aligned using HISAT2 software (version 2.21) with Ensembl human (hg38/GRCh38) reference genomes. The mapped fragments were stored and converted to the BAM format by SAMtools (version 1.4). The read counts of each identified gene were quantified by StringTie (version 1.3.3b) and were then used as input to calculate differential gene expression and statistical significance in DESeq2 (version 3.5). In pairwise comparisons, DEGs were defined as genes with fold changes of more than 1.5 and an adjusted *p‐value* of less than 0.05.

### Enrichment Analysis

Based on the DEGs, Gene Ontology (GO) biological process term enrichment analysis was performed using the Metascape online tool and clusterProfiler R package (version 3.12.0). Kyoto Encyclopedia of Genes and Genomes (KEGG) pathway enrichment analysis was performed by hypergeometric testing with the in‐house Perl script. Gene set enrichment analysis (GSEA) was performed on the Java GSEA platform (version 3.0) based on gene sets defined by the GO and KEGG results. The “Signal2Noise” metric was used to generate a ranked list and permutation type of the gene set. Gene sets with a *p* value of less than 0.05 and a false discovery rate (FDR) value of less than 0.25 were considered statistically significant.

### Public Data Analysis

The bulk RNA‐seq and ChIP‐Seq datasets used in this study were collected and downloaded from GEO database. In the analysis of potential key regulators in vascular injury, three bulk RNA‐seq datasets in mice numbered GSE40637 (control versus injured), GSE119549 (control versus injured_6 h and control versus injured_24 h), and GSE70410 (non‐ligated versus ligated) were collected. The RNA‐seq datasets related to macrophage polarization numbered GSE86298, GSE99296, GSE106700, and GSE158094 were analyzed. In addition, eleven ChIP‐Seq datasets were used for analysis. The Estimate the Proportion of Immune and Cancer Cells (EPIC) algorithm was applied to estimate the proportion of infiltrated cell types in each dataset. The DEGs were identified by the R software package “Limma”. Transcription factors were predicted by the iRegulon plugin of Cytoscape software (version 3.7.2).

### Statistical Analysis

All data were analyzed by SPSS software (version 19.0) and are presented either as the mean ± standard deviation (SD) or median ± interquartile range. For data with a normal distribution, a two‐tailed unpaired Student's t‐test was employed to compare differences between two groups, and one‐way ANOVA was applied for multiple comparisons, followed by Bonferroni's post hoc test (for data meeting homogeneity of variance) or Tamhane's T2 post hoc test (for data showing heteroscedasticity). For data showing a skewed distribution, the Mann‐Whitney U test was used to analyze differences between the two groups, and the Kruskal‐Wallis H test was applied for multiple comparisons. For each experiment, the sample size is indicated in the figure legends. Age‐ and weight‐matched male animals were randomly assigned to groups. Data from animal studies were collected in a blinded fashion. *p* values less than 0.05 were considered significant.

## Conflict of Interest

The authors declare no conflict of interest.

## Author Contributions

J.M., Y.Y., Z.Z., and K.Z. contributed equally to this work. P.Z. and J.C. conceptualized the study. J.M., Y.Y., Z.Z., K.Z., W.L., J.L., and S.Z. designed and performed experiments. W.L. and J.L. developed methodology. J.M., Y.Y., J.‐J.Q., H.S., and Y.W. analyzed and organized the data. X.F. and X.L. validated the data. S.C. and Z.‐G.S. provided resources. J.M., Y.Y., and K.Z. wrote the original draft. P.Z., J.C., and Z.Z. reviewed and edited the manuscript.

## Supporting information



Supporting Information

## Data Availability

The data that support the findings of this study are available from the corresponding author upon reasonable request.
