# Supplementary material for: Artesunate Inhibits Neointimal Hyperplasia by Promoting IRF4 Associated Macrophage Polarization
Source: Adv Sci (Weinh). 2025 Mar 24;12(19):2408992. doi: 10.1002/advs.202408992 (PMC12097016; doi:10.1002/advs.202408992)
Supplement: Supplementary file 1 — Supporting Information [file ADVS-12-2408992-s001.docx]

**Supporting Information**

**Artesunate Inhibits Neointimal Hyperplasia by Promoting IRF4 Associated Macrophage Polarization**

Jinlin Miao, Yule Yong, Zhaohui Zheng, Kui Zhang, Wei Li, Jiayi Liu, Siyi Zhou, Juan-juan Qin, Haoyang Sun, Yatao Wang, Xianghui Fu, Xing Luo, Siyu Chen, Zhi-Gang She, Jingjing Cai,* Ping Zhu*

*Corresponding author. Email: zhuping@fmmu.edu.cn; caijingjing83@hotmail.com

Jinlin Miao, Yule Yong, Zhaohui Zheng, and Kui Zhang contributed equally to this work.

**Appendix Figures**


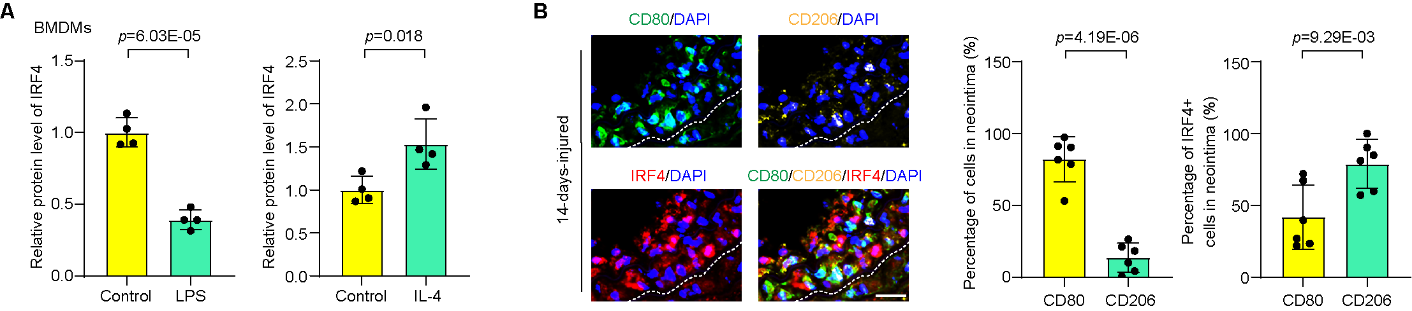


**Figure S1. IRF4 expression in macrophages and neointima of mouse arteries post-injury. (A)**  Quantitative analysis of the protein levels of IRF4 in mouse BMDMs upon LPS and IL-4 treatment. n = 4 per group. **(B)** Multiplex immunohistochemistry co-staining of CD80 (green), CD206 (yellow) and IRF4 (red) in sections of mouse wire-induced carotid neointima at day 14 post-injury. Scatter plot showing the percentage of CD80-positive and CD206-positive cells among nucleated cells in neointima (left), and the percentage of IRF4-positive cells among CD80-positive or CD206-positive cells in neointima (right). n = 6 per group. Scale bar = 25 μm. Data information: The data with error bars are presented as mean ± SD. Two-tailed Student’s t-test is used.


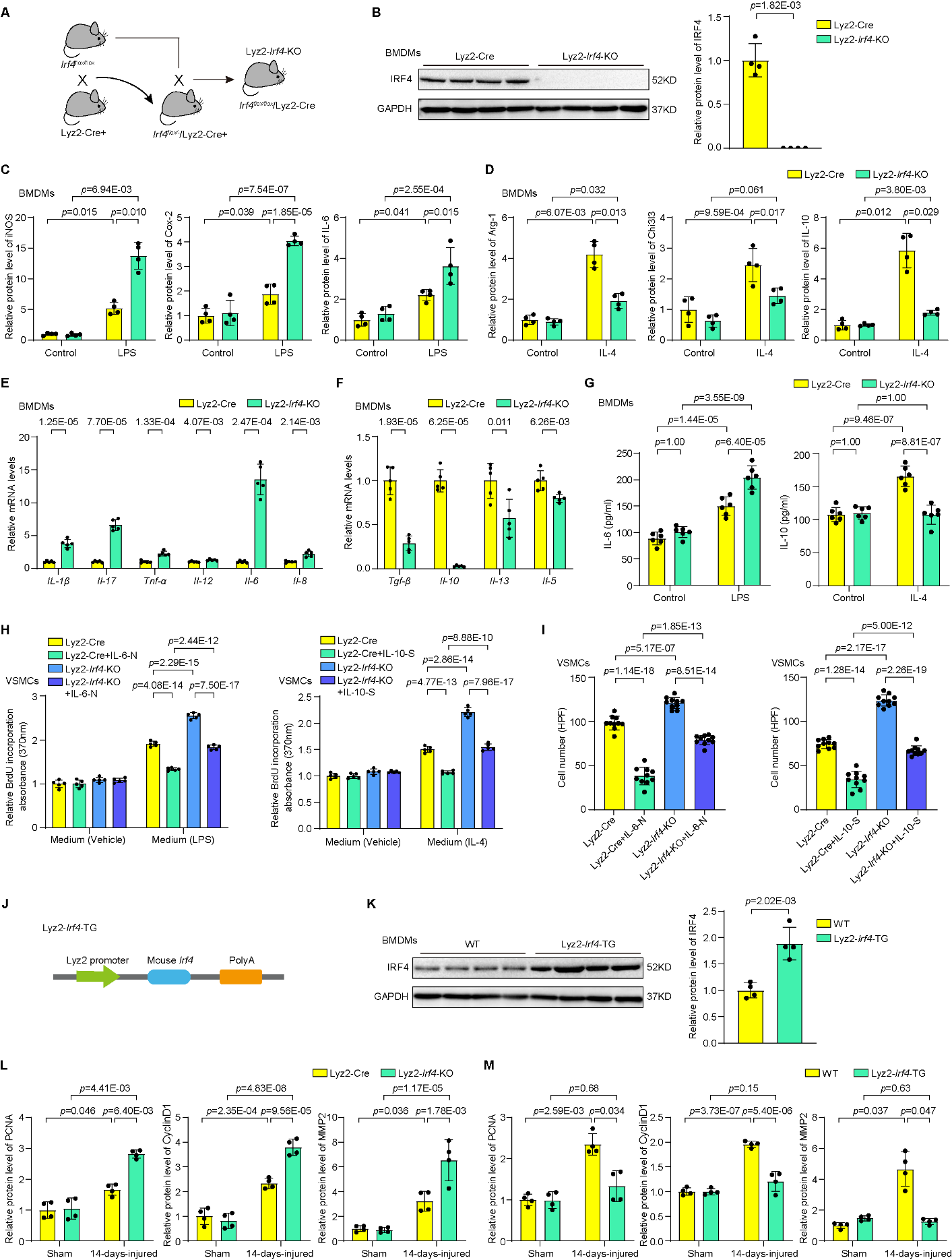


**Figure S2.** **Establishment and functional evaluation of myeloid-specific *Irf4* knockout and transgenic mice.** (**A**) Schematic diagram of the establishment of myeloid-specific *Irf4* knockout (Lyz2-*Irf4*-KO) mice. (**B**) Immunoblotting analysis of IRF4 expression in the BMDMs of Lyz2-Cre and Lyz2-*Irf4*-KO mice. n = 4 per group. (**C** and **D**) Quantitative analysis of the protein levels of M1 macrophage (C) or M2 macrophage (D) markers in mouse BMDMs from Lyz2-Cre and Lyz2-*Irf4*-KO mice upon LPS and IL-4 treatment. n = 4 per group. (**E and F**) Relative mRNA expression levels of cytokines in BMDMs of Lyz2-Cre and Lyz2-*Irf4*-KO mice upon LPS (E) and IL-4 (F) treatment. n = 5 per group. **(G)** Measurement of IL-6 and IL-10 concentrations in the medium of cultured BMDMs of Lyz2-Cre and Lyz2-*Irf4*-KO mice upon LPS or IL-4 treatment by ELISA. n = 6 per group. **(H)** VSMC proliferation was assessed using BrdU incorporation in conditioned medium from Lyz2-Cre and Lyz2-*Irf4*-KO BMDMs, treated with or without IL-6-N (LPS) and IL-10-S (IL-4). n = 5 per group. **(I)** VSMC migration was evaluated by Transwell assay using conditioned medium from Lyz2-Cre and Lyz2-*Irf4*-KO BMDMs, treated with or without IL-6-N (LPS) and IL-10-S (IL-4). n = 10 per group. (**J**) Schematic diagram of the establishment of Lyz2-*Irf4*-TG mice harboring full-length mouse IRF4 cDNA under the control of the Lyz2 promoter. (**K**) Immunoblotting analysis of IRF4 expression in the BMDMs of WT and Lyz2-*Irf4*-TG mice. n = 4 per group. (**L** and **M**) Quantitative analysis of the protein levels of PCNA, CyclinD1 and MMP2 in the arteries of Lyz2-*Irf4*-KO mice (G) and Lyz2-*Irf4*-TG mice (H) after sham or at 14 days post-injury. n = 4 per group. Data information: The data with error bars are presented as mean ± SD. One-way ANOVA followed by Bonferroni’s test (C, D, G, H, I, L, M) or Tamhane’s T2 test (C, D, L, M) and Two-tailed Student’s t-test (B, E, F, K) are used.


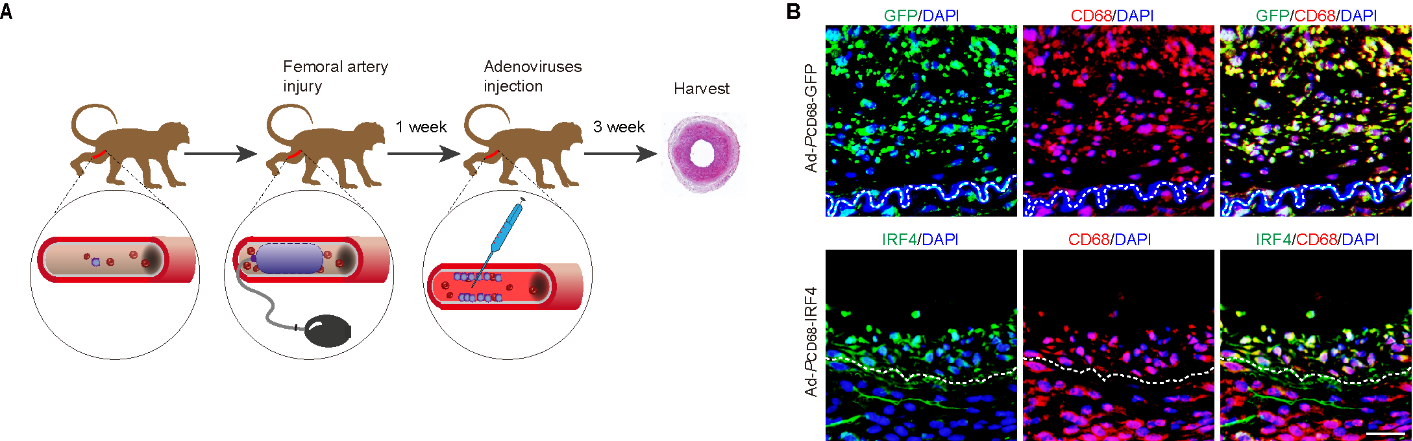


**Figure S3. Strategy of IRF4 adenovirus transfection after artery injury in nonhuman primates.** (**A**) Schematic diagram illustrating the construction of the monkey femoral artery injury model with adenovirus injection. (**B**) Immunofluorescence co-staining of IRF4 (green) with CD68 (red) in arteries from Ad-*P*CD68-GFP and Ad-*P*CD68-IRF4 monkeys. Scale bar = 50 μm.


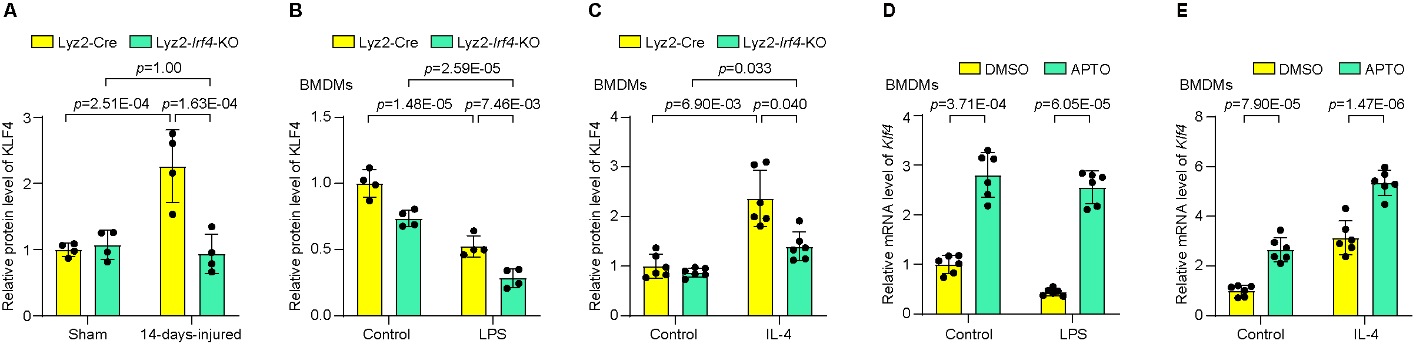


**Figure S4. KLF4 expression after injury or stimulation.** (**A**) Quantitative analysis of the protein levels of KLF4 in the arteries of Lyz2-Cre and Lyz2-*Irf4*-KO mice after sham or at 14 days post-injury. n = 4 per group. (**B** and **C**) Quantitative analysis of the protein levels of KLF4 in BMDMs from Lyz2-Cre and Lyz2-*Irf4*-KO mice upon LPS (B) or IL-4 (C) treatment. n = 4 or 6 per group. (**D and E**) Relative mRNA expression of KLF4 in BMDMs upon DMSO or APTO-253 (APTO) stimulation under LPS (D) or IL-4 (E) treatment. n = 6 per group. Data are presented as the mean ± SD. Data information: The data with error bars are presented as mean ± SD. One-way ANOVA followed by Bonferroni’s test (A, B, E) or Tamhane’s T2 test (C, D) are used.


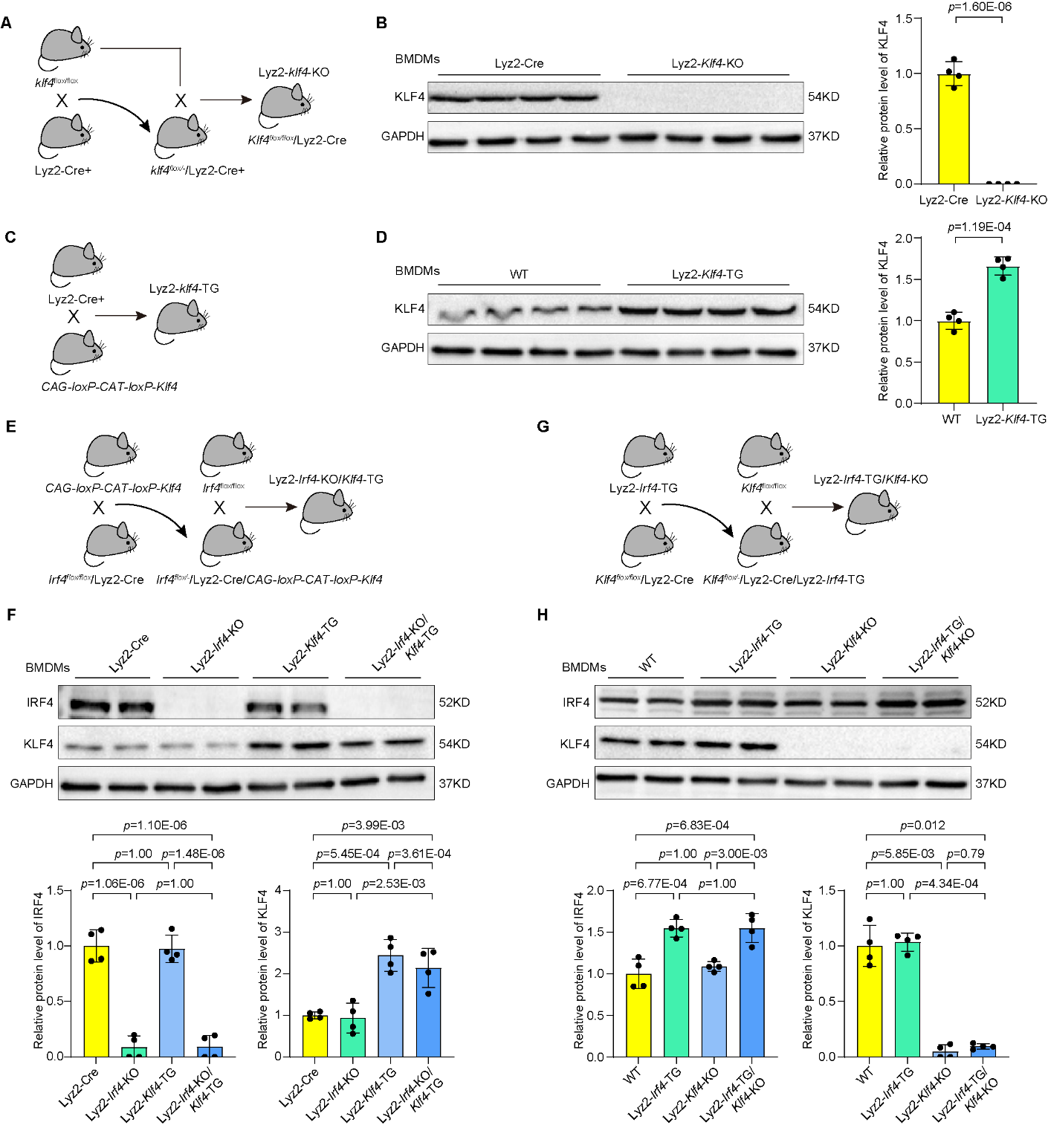


**Figure S5. Establishment of myeloid-specific *Klf4* and combined *Irf4* transgenic mice.** (**A**) Schematic diagram of the establishment of myeloid-specific *Klf4* knockout mice (Lyz2-*Klf4*-KO). (**B**) Immunoblotting analysis of KLF4 protein in BMDMs from Lyz2-Cre and Lyz2-*Klf4*-KO mice. n = 4 per group. (**C**) Schematic diagram of the establishment of myeloid-specific *klf4* transgenic mice (Lyz2-*klf4*-TG). (**D**) Immunoblotting analysis of KLF4 protein in BMDMs from WT and Lyz2-*Klf4*-TG mice. n = 4 per group. (**E**) Schematic diagram of the establishment of myeloid-specific Lyz2-*Irf4*-KO/*Klf4*-TG mice. (**F**) Immunoblotting analysis of IRF4 and KLF4 protein in BMDMs from Lyz2-*Irf4*-KO/*Klf4*-TG mice. n = 4 per group. (**G**) Schematic diagram of the establishment of myeloid-specific Lyz2-*Irf4*-TG/*Klf4*-KO mice. (**H**) Immunoblotting analysis of IRF4 and KLF4 protein in BMDMs from Lyz2-*Irf4*-TG/*Klf4*-KO mice. n = 4 per group. Data information: The data with error bars are presented as mean ± SD. One-way ANOVA followed by Bonferroni’s test (F, H) or Tamhane’s T2 test (H) and Two-tailed Student’s t-test (B, D) are used.


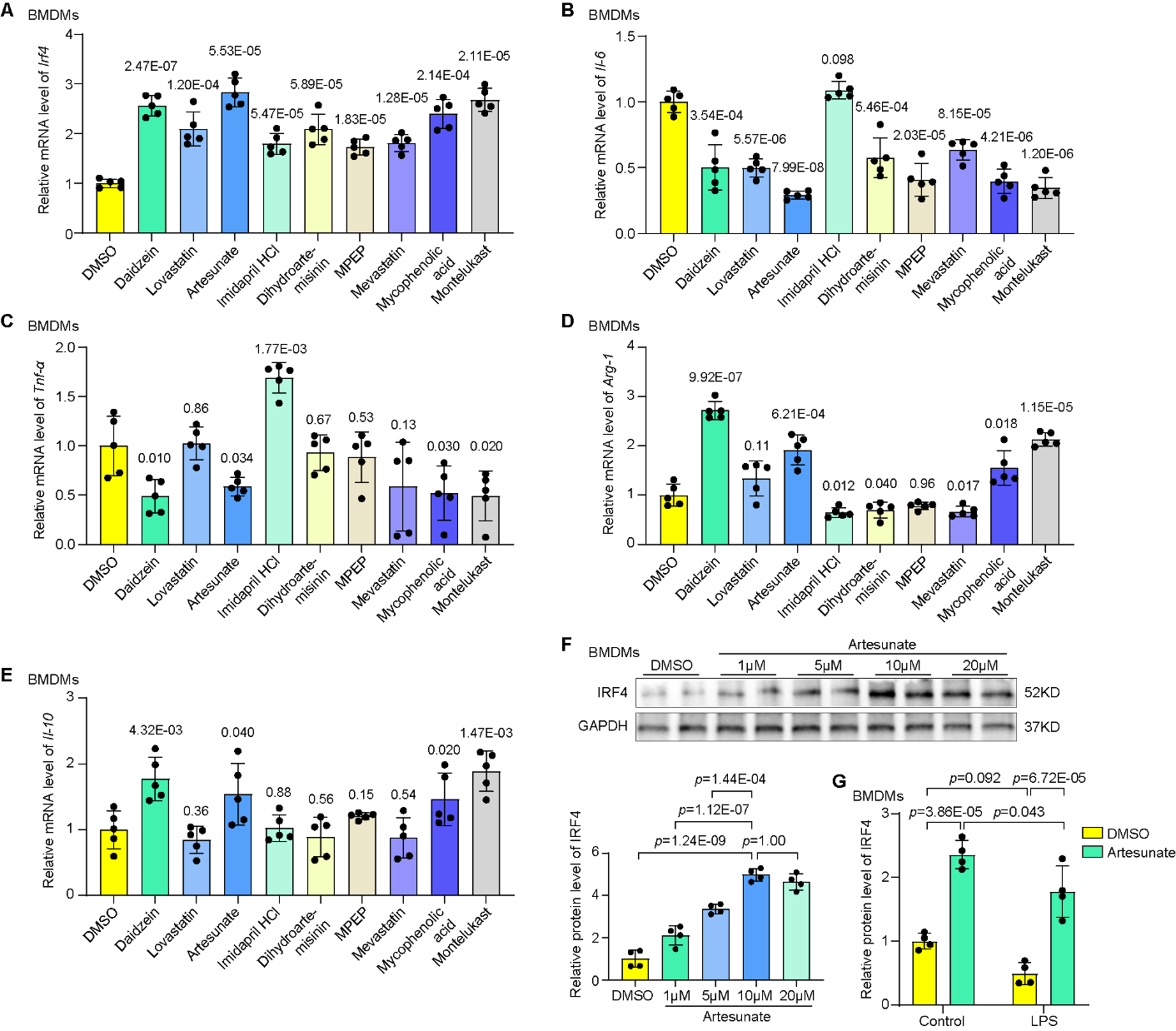


**Figure S6. Regulatory effects of screened drugs on macrophage polarization and IRF4 expression.** (**A**) Relative mRNA expression levels of *Irf4* in BMDMs upon stimulation with 9 screened compounds. n = 5 per group. *p* value *vs.* dimethyl sulfoxide (DMSO) group. (**B, C**) Relative mRNA expression levels of *Il-6* and *Tnf-α* in LPS-treated BMDMs upon stimulation with 9 screened compounds. n = 5 per group. *p* value vs. DMSO group. (**D, E**) Relative mRNA expression levels of *Arg-1* and *Il-10* levels in IL-4-treated BMDMs upon stimulation with 9 screened compounds. n = 5 per group. *p* value vs. DMSO group. (**F**) Immunoblotting analysis of IRF4 protein levels in BMDMs stimulated with DMSO or artesunate for the indicated concentrations. n = 4 per group. (**G**) Quantitative analysis of the protein levels of IRF4 in LPS-treated BMDMs upon DMSO or artesunate (10 μM) stimulation. Data information: The data with error bars are presented as mean ± SD. Two-tailed Student’s t-test (A-E) and One-way ANOVA followed by Bonferroni’s test (F, G) are used.


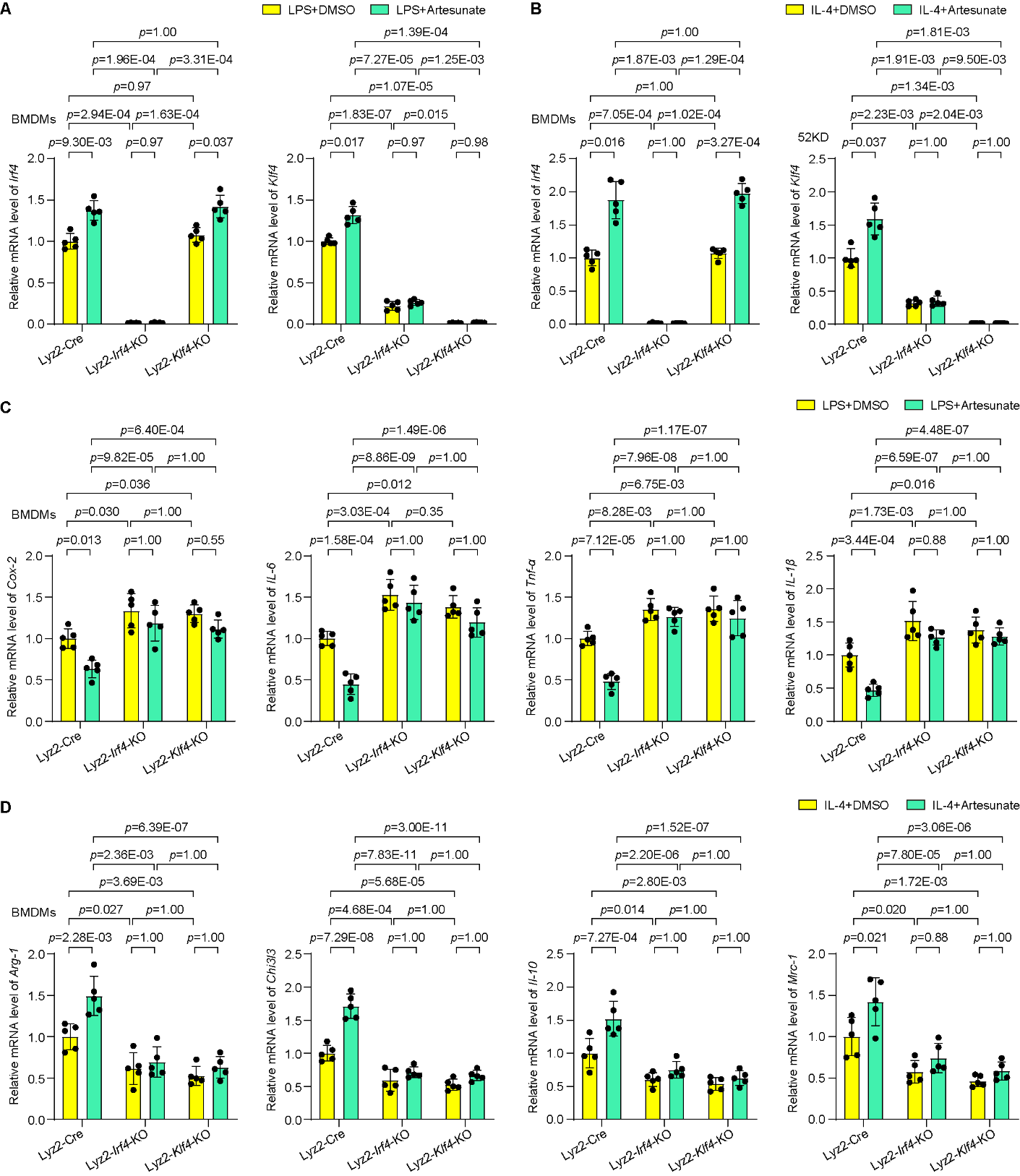


**Figure S7. Regulatory effects of artesunate on macrophage polarization.** (**A, B**) Relative mRNA expression levels of *Irf4* and *Klf4* in LPS-treated (A) and IL-4-treated (B) BMDMs from Lyz2-*Irf4*-KO and Lyz2-*Klf4*-KO mice upon DMSO or artesunate stimulation. n = 5 per group. (**C**) Relative mRNA expression levels of *Cox-2*, *Il-6*, *Tnf-α*, and *Il-β* in LPS-treated BMDMs from Lyz2-*Irf4*-KO and Lyz2-*Klf4*-KO mice upon DMSO or artesunate stimulation. n = 5 per group. (**D**) Relative mRNA expression levels of *Arg-1*, *Chi3l3*, *Il-10*, and *Mrc-1* in IL-4-treated BMDMs from Lyz2-*Irf4*-KO and Lyz2-*Klf4*-KO mice upon DMSO or artesunate stimulation. n = 5 per group. Data information: The data with error bars are presented as mean ± SD. One-way ANOVA followed by Bonferroni’s test (A, B, C, D) or Tamhane’s T2 test (B) are used.


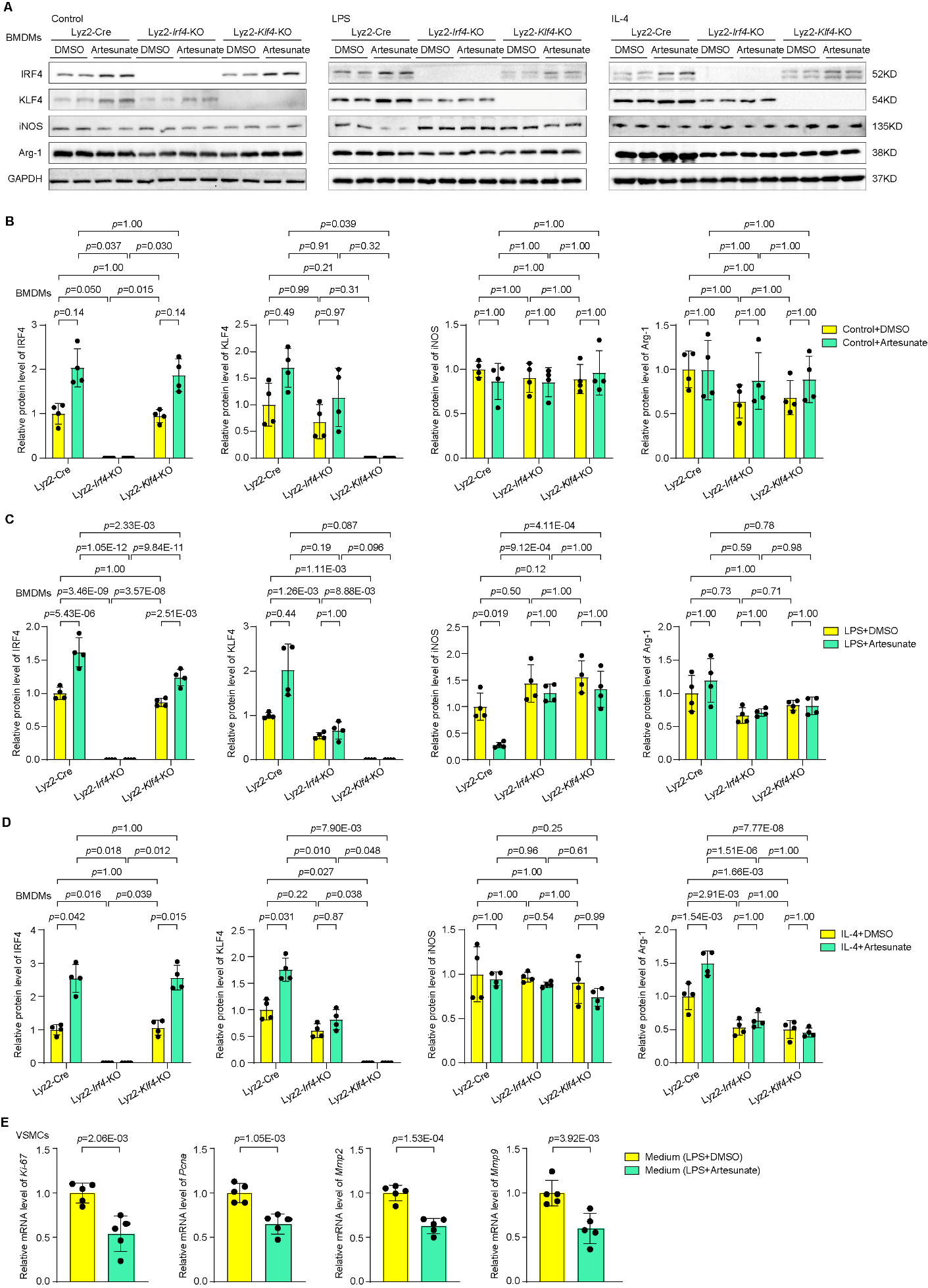


**Figure S8. Regulatory effects of artesunate on macrophage and VSMCs.** (**A** to **D**) Immunoblotting analysis of IRF4, KLF4, iNOS, and Arg-1 protein levels in LPS-treated and IL-4-treated BMDMs from Lyz2-*Irf4*-KO and Lyz2-*Klf4*-KO mice upon DMSO or artesunate stimulation. n = 5 per group. (**E**) Relative mRNA expression levels of *Ki-67*, *Pcna*, *Mmp2*, and *Mmp9* in VSMC cocultured with conditioned medium from LPS-treated BMDMs stimulated with DMSO or artesunate. n = 5 per group. Data information: The data with error bars are presented as mean ± SD. One-way ANOVA followed by Bonferroni’s test (B, C, D) or Tamhane’s T2 test (B, C, D) and Two-tailed Student’s t-test (E) are used.


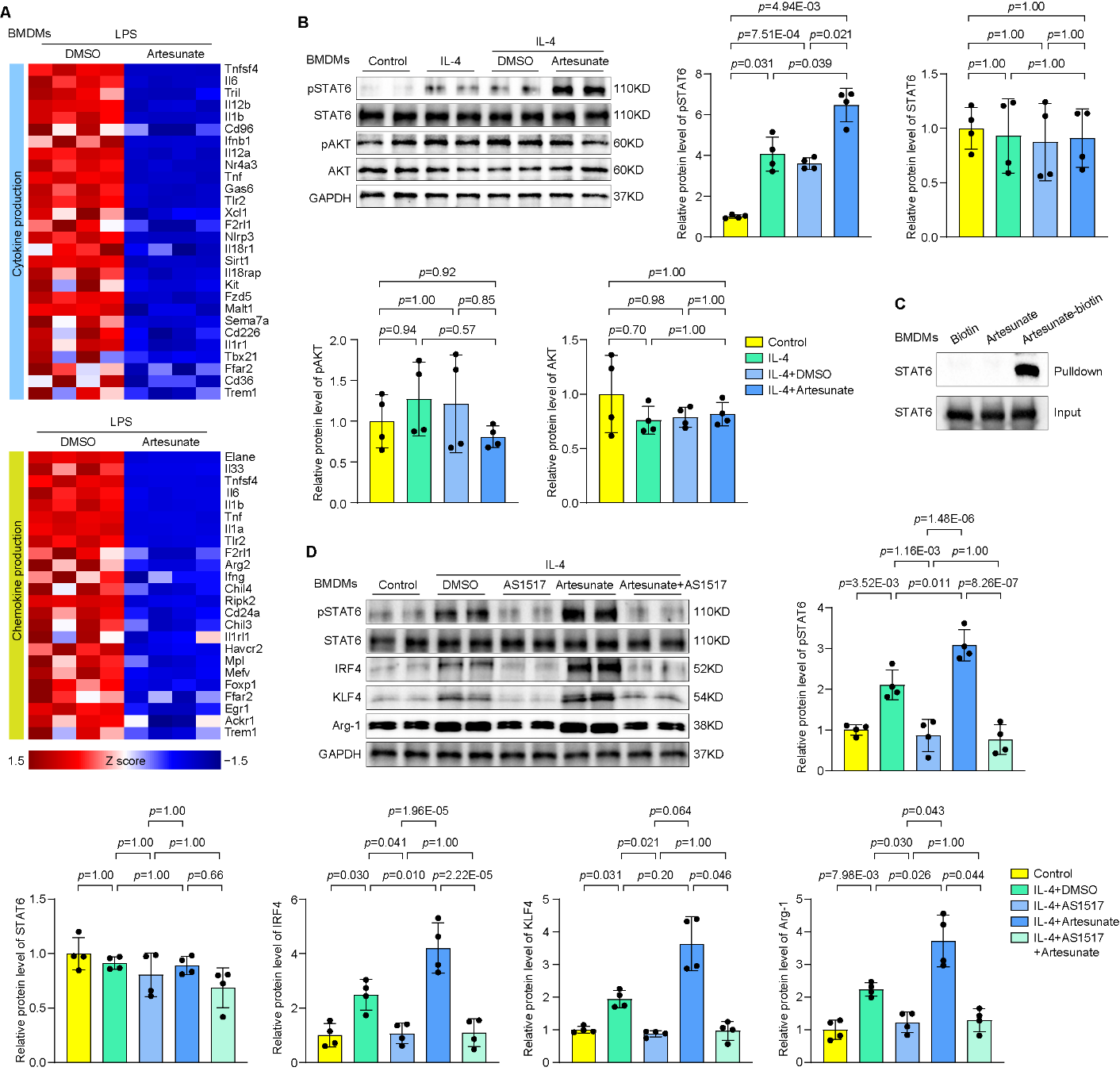


**Figure S9. Artesunate regulates inflammation-related genes and signal molecules in BMDMs. (A)** In LPS-treated BMDMs treated with DMSO or artesunate, heatmaps showing the expression profile of genes related to cytokine production (upper panel) and chemokine production (lower panel) based on RNA-seq data. n = 4 per group. **(B)** Immunoblotting analysis of STAT6, pSTAT6 (Tyr641), AKT, and pAKT (Ser473) protein levels in IL-4-treated BMDMs stimulated with DMSO or artesunate. n = 4 per group. **(C)** BMDMs from mice were treated with biotin, artesunate, or artesunate-biotin probes, then the proteins were pulled down using streptavidin magnetic beads, and the STAT6 protein level was detected by Western blotting assay. **(D)** BMDMs from mice were pretreated with or without 100 nM STAT6 inhibitor AS1517499 for 30 minutes upon DMSO or artesunate stimulation in the presence of IL-4, followed immunoblotting analysis of STAT6, pSTAT6 (Tyr641), IRF4, KLF4, and Arg-1 protein levels. n = 4 per group. AS1517, a small molecule of pSTAT6 inhibitor (AS1517499). Data information: The data with error bars are presented as mean ± SD. One-way ANOVA followed by Bonferroni’s test (B, D) or Tamhane’s T2 test (B, D) are used.

**Appendix Tables**

**Table S1. Demographic and clinical characteristics of human donors**

|  | Patients with  neointima (n=4) | Controls  (n=4) |
| --- | --- | --- |
| Age, years | 61.00 ± 7.79 | 53.25 ± 6.70 |
| Female | 1 (25.00%) | 1 (25.00%) |
| BMI, kg/m^2^ | 25.85 ± 2.70 | 23.59 ± 1.57 |
| Serum creatinine, mg/dl | 1.05 ± 0.31 | 1.13 ± 0.39 |
| Active smokers | 2 (50.00%) | 1 (25.00%) |
| Hypercholesterolemia | 3 (75.00%) | 1 (25.00%) |
| Hypertension | 3 (75.00%) | 2 (50.00%) |
| Diabetes mellitus | 1 (25.00%) | 1 (25.00%) |
| Unstable angina | 1 (25.00%) | NA |
| Previous myocardial infarction | 2 (50.00%) | NA |

Data are presented as mean ± SD or n (%). BMI, body mass index; NA, not available.

**Table S2. Antibodies used in the present study.**

| **Antibody** | **Cat.No.** | **Manufacturer** | **Reactivity** | **Application** |
| --- | --- | --- | --- | --- |
| AKT | 4691 | Cell Signaling Technology | Ms | WB |
| Arg-1 | 610708 | BD Biosciences | Ms | WB, IF |
| α-SMA | ab7817 | Abcam | Ms, Mk | IF |
| CD68 | MA5-13324 | Invitrogen | Mk | IF |
| CD80 | A16039 | Abclonal | Ms | IHC |
| CD206 | 24595 | Cell Signaling Technology | Ms | IHC |
| Chi3l3 | ab93034 | Abcam | Ms | WB |
| Cox-2 | 4842 | Cell Signaling Technology | Ms | WB |
| CyclinD1 | 2978 | Cell Signaling Technology | Ms | WB |
| GAPDH | 2118 | Cell Signaling Technology | Ms | WB |
| IL-6 | AF-406-NA | R&D Systems | Ms | WB |
| IL-6 | SC-1265 | Santa Cruz Biotechnology | Ms | IF |
| IL-6 | MA5-23698 | Invitrogen | Mk | IF |
| IL-10 | AF519 | R&D Systems | Ms | WB |
| iNOS | ab3523 | Abcam | Ms | WB |
| iNOS | A3774 | Abclonal | Ms | IF |
| IRF4 | 06-1047 | Millipore | Hu, Ms | WB, IF, IHC |
| IRF4 | PA5-115603 | Invitrogen | Mk | IF |
| Ki-67 | ab15580 | Abcam | Ms, Mk | IF |
| KLF4 | ab151733 | Abcam | Ms | WB |
| Mac3 | 550292 | BD Biosciences | Ms | IF |
| Mac3 | ab25631 | Abcam | Hu, Mk | IF |
| MMP2 | sc13595 | Santa Cruz Biotechnology | Ms | WB |
| MMP2 | ab92536 | Abcam | Ms | IF |
| MMP2 | 436000 | Invitrogen | Mk | IF |
| pAKT | 4060 | Cell Signaling Technology | Ms | WB |
| PCNA | 2586 | Cell Signaling Technology | Ms | WB |
| PPARγ | ab209350 | Abcam | Ms | IF |
| pSTAT6 | 56554 | Cell Signaling Technology | Ms | WB |
| STAT6 | 5397 | Cell Signaling Technology | Ms | WB |

Cat.No., Catalog Number; Hu, human; Mk, monkey; Ms, mouse; IF, immunofluorescence; WB, western blot; IHC, immunohistochemistry.

**Table S3. The primers for real-time PCR.**

| **Primer** | **Sequence (5’ to 3’)** | **GenBank** |
| --- | --- | --- |
| *Irf4*-F | GCCAGCCCAGGTTCATAACTA | NM_001347508.1 |
| *Irf4*-R | AGGTGGGGCACAAGCATAA |  |
| *Klf4*-F | GCCACCCACACTTGTGACTA | XM_036163748.1 |
| *Klf4*-R | CTGTGTGTTTGCGGTAGTGC |  |
| *Tnf-α*-F | ATGGCCTCCCTCTCATCAGT | NM_001278601.1 |
| *Tnf-α*-R | ATAGCAAATCGGCTGACGGT |  |
| *Il-6*-F | CTGGAGTACCATAGCTACCTGG | NM_001314054.1 |
| *Il-6*-R | ACTCCTTCTGTGACTCCAGC |  |
| *Cox-2*-F | ATTCCCTCCGGTGTTTGTCC | NM_011198.5 |
| *Cox-2*-R | TAGGCTTTGCTGGCTACCAC |  |
| *Inos*-F | AGGGCCACCTCTACATTTGC | NM_010927.4 |
| *Inos*-R | TGCCCCATAGGAAAAGACTGC |  |
| *Chi3l3*-F | AGGGCCCTTATTGAGAGGAG | NM_009892.4 |
| *Chi3l3*-R | AGCTGGTACAGCAGACAAGAC |  |
| *Arg-1*-F | AAAGGCCGATTCACCTGAGC | NM_007482.3 |
| *Arg-1*-R | AGGTAGTCAGTCCCTGGCTT |  |
| *Il-10*-F | CATCGATTTCTCCCCTGTGA | NM_010548.2 |
| *Il-10*-R | CATTCATGGCCTTGTAGACACC |  |
| *Mrc-1*-F | CCTGTAACTACACACTCATCCA | NM_008625.2 |
| *Mrc-1*-R | CATTCTGCTCGATGTTGCCC |  |
| *Il-1β*-F | TAATGAAAGACGGCACACCCA | NM_008361.4 |
| *Il-1β*-R | GTTTCCCAGGAAGACAGGCT |  |
| *Ki-67*-F | ACCATCATTGACCGCTCCTTT | NM_001081117.2 |
| *Ki-67*-R | AGGCCCTTGGCATACACAAA |  |
| *Pcna*-F | GAACCTCACCAGCATGTCCA | NM_011045.2 |
| *Pcna*-R | ATTCACCCGACGGCATCTTT |  |
| *Ccl2*-F | CAGGTCCCTGTCATGCTTCT | NM_011333.3 |
| *Ccl2*-R | GAGTGGGGCGTTAACTGCAT |  |
| *Cxcl2*-F | AGGGCGGTCAAAAAGTTTGC | NM_009140.2 |
| *Cxcl2*-R | CGAGGCACATCAGGTACGAT |  |
| *Mmp2*-F | TCCCTAAGCTCATCGCAGAC | XM_006530751.4 |
| *Mmp2*-R | GGCTGCTTCACATCCTTCAC |  |
| *Mmp9*-F | CGCTCATGTACCCGCTGTAT | NM_013599.5 |
| *Mmp9*-R | CCGTGGGAGGTATAGTGGGA |  |
| *Gapdh*-F | GAAGGGCATCTTGGGCTACA | NM_001411843.1 |
| *Gapdh*-R | GGGGTCTGGGATGGAAATTGT |  |
